# Research Progress and Prospects of Ultra-High-Temperature Ceramics: Experimentation, Multiscale Simulation and Data-Driven Design

**DOI:** 10.3390/nano16110693

**Published:** 2026-06-01

**Authors:** Nan Qu, Wentao Zhou, Wei Zhang, Yong Liu, Lu Zheng, Dingbo Cao, Mingyi Tan, Jingchuan Zhu, Xinghong Zhang

**Affiliations:** 1 School of Materials Science and Engineering, Harbin Institute of Technology, Harbin 150001, China; 2 Science and Technology on Advanced Composites in Special Environments Laboratory, Harbin Institute of Technology, Harbin 150001, China

**Keywords:** ultra-high-temperature ceramics, high-entropy ceramics, oxidation resistance, structure–property relationships, computational materials science

## Abstract

Ultra-high-temperature ceramics (UHTCs), including transition-metal carbides, nitrides, and diborides, have emerged as a class of promising structural materials for applications in extreme aerospace and energy environments. Their strong covalent–metallic bonding endows them with exceptionally high melting points, elastic moduli, and thermal stability. Nevertheless, intrinsic brittleness, limited oxidation resistance, and poor sinterability remain key challenges for the engineering application of conventional UHTCs. Recently, novel material design strategies such as multiphase composites, microstructural engineering, and compositional complexity have emerged. Among these, high-entropy UHTCs (HE-UHTCs) have attracted significant attention due to their configurational entropy, lattice distortion, and sluggish diffusion effects, which collectively enhance oxidation resistance, thermal stability, sinterability, and mechanical performance. This review summarizes the crystal chemistry, mechanical behavior, oxidation, and ablation properties of conventional UHTCs and HE-UHTCs. The four core effects of HE-UHTCs—configurational entropy, lattice distortion, sluggish diffusion, and cocktail effects—are discussed in relation to their mechanical properties and oxidation resistance. The roles of computational materials science, including density functional theory (DFT), molecular dynamics (MD), and machine learning, in composition screening and property prediction are critically reviewed. Finally, key challenges and future directions for the rational design and engineering application of UHTCs are discussed.

## 1. Introduction

The rapid advancement of aerospace technologies, particularly hypersonic vehicles and rocket propulsion systems, imposes increasingly stringent requirements on thermal protection materials. Critical components, such as nose cones, leading edges, combustion chambers, and nozzles, must withstand extreme service environments characterized by temperatures exceeding 2000 °C, high-velocity gas erosion, severe thermal shock, and oxygen-rich atmospheres, while maintaining dimensional stability and reliable load-bearing capacity. Conventional high-temperature alloys suffer from significant strength degradation at elevated temperatures and are therefore inadequate for such demanding conditions. Conventional ceramics also face oxidation failure and thermal-shock sensitivity at high temperatures.

UHTCs have emerged as a class of extreme-environment structural materials addressing these challenges. From the perspective of intrinsic material properties, the advantages of UHTCs arise not only from their high melting points but also from their transition-metal–nonmetal bonding characteristics. Typical UHTCs generally have melting points above 3000 °C, and some systems, such as Hf(C,N), can reach approximately 4200 °C. They also exhibit high thermal conductivity (more than 140 W·m^−1^·K^−1^) and high elastic modulus (up to 600 GPa) [1]. This mixed covalent–metallic bonding enables UHTCs to maintain structural stability under extreme thermal, mechanical, and chemical environments [2].

Despite these advantages, UHTCs face significant engineering challenges. First, the strong directional covalent bonding makes the material inherently brittle and prone to failure under thermal mechanical coupling. Second, in the high-temperature oxidation environment, the surface oxide layer may volatilize or become porous, making it difficult to provide long-term protection. Third, low self-diffusion coefficients and strong bonding make densification difficult and increase processing costs. Although the composite design, second-phase collaborative optimization and microstructure design have made gradual progress, these strategies have not yet fully overcome the performance limitations of UHTCs under coupled extreme environments.

HE-UHTCs achieve entropy-driven phase stabilization by introducing a variety of near-equiatomic components into the same lattice, providing a promising strategy for overcoming the performance limitations of conventional UHTCs. HE-UHTCs use configurational entropy to stabilize high-temperature single-phase solid solutions. Together with severe lattice distortion and sluggish diffusion, entropy-driven stabilization significantly alters phase stability, diffusion kinetics, oxidation behavior, and mechanical response. Therefore, HE-UHTCs exhibit stronger oxidation resistance, better thermal stability and better sinterability than traditional ceramics.

This review provides a comprehensive assessment of conventional and high-entropy UHTCs. We also highlight how computational materials science and machine learning facilitate exploration of vast compositional spaces, enabling rational design of UHTCs for extreme environments.

## 2. Typical UHTC Systems and Crystal Structure Characteristics

### 2.1. Crystal Structures of UHTCs

**Figure 1:**
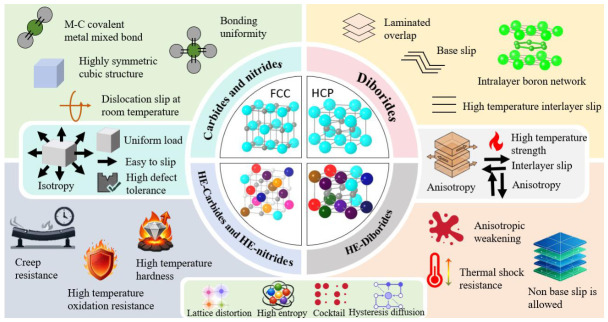
Crystal structures, bonding characteristics, and representative properties of ultra-high-temperature carbides, nitrides, and diborides.

UHTCs mainly include transition-metal carbides, nitrides and diborides. Although UHTCs exhibit ultra-high melting points and excellent thermal stability at the macroscopic scale, fundamental differences exist in their crystal topology, bonding directionality, and defect tolerance. These structural characteristics ultimately determine their mechanical response, thermal transport behavior, and failure mechanisms under extreme service conditions. Figure 1 introduces the types, crystal structures, and related properties of UHTCs.

Transition-metal carbides and nitrides typically crystallize in the NaCl-type face-centered cubic (FCC) structure. In this structure, metal atoms form a densely packed FCC lattice, while nonmetal atoms occupy octahedral interstitial sites. The high symmetry and uniform atomic packing of this structure result in nearly isotropic mechanical and thermal responses at the macroscopic scale. Such structural features facilitate homogeneous load transfer at high temperatures and enhance tolerance to point defects and deviations from stoichiometry.

However, due to the relatively limited bond strength of metal–nonmetal (M-X) bonds, the high-temperature strength of NaCl-type UHTCs is generally lower than that of layered diboride systems, especially under ultra-high-temperature and high-stress conditions.

From the perspective of plastic deformation mechanisms, even within the same crystal structure, different transition-metal carbides still show significantly different dislocation slip behavior. For example, TaC preferentially deforms plastically through the {111}<110>slip system. This behavior is closely related to stable intrinsic stacking faults on {111} planes, which reduce the energy barrier for dislocation nucleation and motion. In contrast, HfC tends to deform on the {110}<110>slip system and lacks internal stacking faults, resulting in a significant increase in slip resistance [3,4,5,6].

This difference reflects a general trend in which group-VB carbides typically possess intrinsic stacking faults, whereas group-IVB carbides do not. Consequently, these materials display systematic differences in low-temperature plasticity and high-temperature creep behavior [7]. These observations highlight that microscopic defect structures can significantly influence the mechanical response of UHTCs even within identical crystal structure.

In contrast to carbides and nitrides, transition-metal diborides usually adopt the AlB_2_-type hexagonal structure. This structure consists of alternating layers of metal atoms and two-dimensional boron honeycomb networks [2]. Strong covalent bonding occurs between boron atoms, whereas metal–boron bonds exhibit mixed covalent–metallic character.

The layered structure leads to pronounced anisotropy in mechanical and transport properties. Within the basal plane (a-direction), the continuous covalent boron network provides extremely high stiffness and load-bearing capacity. Along the c-axis, weaker interlayer bonding results in reduced elastic modulus and fracture resistance. For instance, the elastic modulus of TiB_2_ reaches approximately 671 GPa in the a-direction but decreases to about 473 GPa along the c-axis [2].

With increasing transition-metal valence electron concentration, the directionality and covalent character of metal–boron bonds are further enhanced. This bond strengthening further increases the anisotropy of diboride materials [2]. Although such structural characteristics contribute to high-temperature stability and strength, they may also intensify thermal stress concentration and promote preferential crack propagation.

Additionally, strong metallic interactions between boron layers facilitate electron transport within the basal plane, leading to relatively high electrical and thermal conductivity in this direction. This structure-dominated anisotropic transport gives diborides excellent high-temperature mechanical properties and thermal conduction along specific crystallographic directions, but also imposes stricter requirements on microstructural design and orientation-dependent service conditions.

### 2.2. Bonding–Property Relationships of UHTCs

The exceptional melting points and thermal stability of UHTCs originate from the strong interactions between transition-metal d orbitals and nonmetal p orbitals. From the atomic scale, the structural stability and multiphysical properties of UHTCs come from three basic interactions: metal–nonmetal (M–X), metal–metal (M–M), and nonmetal–nonmetal (X–X) bonding.

Among these interactions, M–X bonding exhibits both ionic and covalent character and plays a dominant role in determining melting temperature and high-temperature strength. M–M bonding is primarily metallic and contributes significantly to electronic and thermal conductivity. The X–X bond (especially in boride) has a decisive effect on the structural stiffness and anisotropic response.

The differences in bonding configurations of different UHTC systems directly lead to systematic differences in melting point, thermal conductivity and mechanical properties. Diborides often exhibit higher thermal conductivity than carbides and nitrides due to stronger metallic bonding interactions. In contrast, carbides typically display superior mechanical stability at ultra-high temperatures because of the strong M–C covalent bonds. The high bonding energy significantly suppresses atomic diffusion and lattice vibrations, enabling these materials to retain high elastic modulus and strength even at elevated temperatures. In diborides, the two-dimensional covalent boron networks provide extremely high in-plane stiffness, while the relatively weaker interlayer bonding leads to pronounced anisotropy in crack propagation paths and fracture behavior. In contrast, carbides and nitrides possess nearly isotropic bonding networks, resulting in more uniform macroscopic mechanical responses.

It is worth noting that many typical ultra-high-temperature ceramics, especially transition-metal borides and some carbides, are often considered to be electron-deficient compounds due to the insufficient number of valence electrons required to satisfy the local covalent bond saturation, resulting in pronounced multicenter bonding or mixed covalent–metallic bonding.

In borides, beyond the layered hexagonal sheets found in AlB_2_-type compounds, boron atoms can also form complex polyhedral structures such as B_6_ octahedra, B_12_ cuboctahedra, and B_12_ icosahedra [1]. Because these polyhedra lack sufficient valence electrons to fully satisfy local bonding requirements, the electronic structure becomes highly sensitive to valence electron concentration [8]. The diversity of structures and bonding characteristics in borides provides opportunities for tailoring the properties of boride-based UHTCs. For example, the incorporation of secondary phases such as Y_2_O_3_ has been shown to improve toughness in certain systems [9]. This feature renders the properties of UHTCs highly sensitive to the valence electron concentration (VEC). As the transition-metal constituents or compositional complexity vary, the covalent character of M–X bonds, the degree of electron localization, and the directionality of bonding can be systematically modified, leading to significant variations in melting point, elastic modulus, and thermal transport behavior. In general, increased electron localization enhances M–X covalency, which tends to increase melting point and elastic modulus while reducing thermal conductivity. From the perspective of electron-deficient compounds, the high melting points and moduli of UHTCs are therefore governed not only by bond strength but also by valence-electron filling and electronic-structure stability.

In carbides such as HfC and TaC, and nitrides such as HfN and TaN, because carbon and nitrogen are more electronegative than transition metals, charge transfer tends to occur toward the nonmetal sublattice. These carbides and nitrides therefore exhibit strong covalent bonding with a certain degree of ionic character. In carbides, nitrides and diborides, the melting point, elastic modulus and other mechanical properties of ultra-high-temperature ceramics are changed by changing metal or nonmetal elements to obtain additional valence electrons to change the M–X bond length [2]. Comparisons of bonding energies among compounds with similar crystal structures but different compositions have led to the general conclusion that an increase in the number of valence electrons tends to reduce the formation energy of UHTC systems and consequently decreases the stability of their crystal structures [2]. For nonstoichiometric carbides such as TiC_x_, ZrC_x_, and HfC_x_, the melting point is governed not only by valence-electron filling but also by carbon-vacancy concentration, M-C bond strength, M-M bonding, defect ordering, configurational entropy, and phase stability.

In addition to composition regulation, nonstoichiometric vacancies and point defects have a profound impact on the performance of UHTCs. The introduction of vacancies breaks the long-range periodicity of bonding in the ideal lattice, and systematically regulates covalency, electron localization and bonding directionality through local electron reconstruction, charge compensation and lattice distortion. For transition-metal carbides, metal atoms adjacent to carbon vacancies move closer to each other and form stronger M–M bonds, and the bond length of M–X bonds around the vacancy will be reduced due to charge compensation [10]. Vacancies introduced by nonstoichiometry act as strong phonon-scattering centers. For example, carbon vacancies in ZrC_x_ enhance phonon and electron scattering, resulting in decreased thermal conductivity and heat capacity as the C/Zr ratio decreases [11]. With the continuous decrease in x, ZrC_x_ may transform from a high-thermal-conductivity material to a thermal insulator [11].

Carbon vacancies also induce nonlinear variations in lattice parameters. The lattice constant of ZrC*_x_* reaches the maximum when *x* is about 0.85 and subsequently decreases with further increases in carbon content. This behavior arises from the structural expansion required to accommodate additional carbon atoms. However, as carbon content continues to increase, the lattice parameter decreases due to changes in bonding characteristics associated with composition variations [12]. Vacancies usually reduce melting points by weakening local bonding; however, in some UHTC systems, vacancy-induced M–M bonding can locally increase bonding strength, leading to nonmonotonic melting-point variations with composition. For example, the maximum melting points of TiC_x_, ZrC_x_, and HfC_x_ occur at relative carbon contents of x = 0.80, 0.82, and 0.94, reaching 3345 K, 3708 K, and 4192 K, respectively [13].

Furthermore, vacancies can be regarded as atomic-scale defects that distort local bonding environments, and the bonding distortion around vacancies makes the interatomic potential asymmetric; therefore, vacancies generally reduce the elastic modulus. First-principles calculations indicate that tuning carbon concentration can significantly improve the mechanical and thermodynamic properties of Ta–C carbides. In Ta_6_C_5_, Ta_8_C_7_, TaC, and Ta_2_C systems, the calculated Vickers hardness and elastic modulus increase with increasing carbon concentration. Ta_6_C_5_, Ta_8_C_7_, and TaC exhibit brittle behavior, whereas Ta_2_C and Ta_4_C_3_ display ductile characteristics. In addition, these Ta–C carbides show excellent elastic isotropy. The calculated thermodynamic stability follows the trend Ta_6_C_5_ > Ta_8_C_7_ > TaC > Ta_2_C [14]. These findings indicate that vacancies do not simply degrade material properties but instead provide a tunable structural design parameter.

Nitrogen vacancies can also significantly influence material performance. One perspective suggests that nitrogen vacancies can serve as pinning centers that inhibit dislocation motion, thereby enhancing the mechanical strength of thin films [15]. Another viewpoint emphasizes that electronic bands and the density of states (DOS) near the Fermi level strongly affect the mechanical rigidity and electronic properties of transition-metal nitride (TMN) films, resulting in anomalous behavior when the number of electrons per unit cell changes [16].

As the concentration of nitrogen vacancies increases, the electronic density near the Fermi level rises, leading to changes in hardness, fracture toughness, and electrical properties. Meanwhile, the lattice parameter decreases with increasing nitrogen vacancy concentration [17].

For hafnium nitride (HfN_x_), substoichiometric Hf_55_N_45_ and near-stoichiometric Hf_51_N_49_ films exhibit nanoindentation hardness values of 24.6 GPa and 26.0 GPa, respectively. As the nitrogen content increases further to 53, 58, and 63 at.%, the hardness decreases to 20.7, 18.6, and 17.8 GPa, respectively. Stress-free Hf_33_N_67_ films exhibit a hardness as low as 7.1 GPa. Young’s modulus decreases monotonically with increasing stoichiometric variable x [18]. ZrN_x_ films with N/Zr ≈ 0.7 show the lowest corrosion rate and best oxidation resistance, whereas those with N/Zr ≈ 0.75 show the poorest corrosion and oxidation resistance [19].

In terms of electrical properties, a phase transition occurs from HfN to Hf_3_N_4_ as the stoichiometric parameter x increases from 1.039 to 1.334, accompanied by an increase in film resistivity of nearly three orders of magnitude, indicating that films with the Hf_3_N_4_ structure are nearly electrically insulating, whereas films with the HfN structure exhibit metallic behavior [20]. Collectively, these studies demonstrate that the properties of UHTCs are not determined solely by their ideal crystal structures but instead arise from the complex interplay among electronic structure, valence electron filling, and defect states.

## 3. Mechanical and Thermal Properties of UHTCs

### 3.1. Mechanical Properties of UHTCs

UHTCs generally exhibit high hardness and elastic modulus due to their strong atomic bonding and dense crystal structures. These characteristics enable them to maintain structural stability under extreme thermomechanical environments. However, similar to many structural ceramics, UHTCs typically suffer from low fracture toughness, which remains a major limitation for engineering applications. At room temperature, ultra-high-temperature carbides typically possess hardness values of 14–30 GPa and fracture toughness values of approximately 2–3.46 MPa·m^1/2^ [21]. Their elastic moduli generally fall within 300–540 GPa [22]. In comparison, ultra-high-temperature nitrides exhibit slightly lower hardness (10–18 GPa) but comparable elastic modulus values of 380–490 GPa. However, nitrides tend to possess relatively high coefficients of thermal expansion and lower thermal conductivity, which to some extent weakens their resistance to thermal shock [23].

From a micromechanistic perspective, pronounced covalent bonding and complex crystal structures strongly inhibit dislocation nucleation and motion in UHTCs. As a result, these materials typically exhibit brittle fracture behavior. This intrinsic brittleness significantly limits the reliability of monolithic UHTCs in extreme service environments, making toughening strategies essential for practical applications [24].

The toughening strategies for UHTCs can be divided into intrinsic mechanisms, such as dislocation plasticity and phase transformation, and extrinsic mechanisms, such as crack bridging and fiber pull-out. In UHTC composites, intrinsic toughening mainly involves dislocation plasticity and phase-transformation effects. Lattice distortion and local stress concentration can be manipulated through grain-size control and compositional optimization to improve toughness. Grain refinement can hinder crack propagation and increase the energy consumed during crack growth. In addition, high dislocation densities may enable localized plasticity at ceramic crystal surfaces [25]. Local dislocation slip and stress concentration can guide crack deflection, generate tortuous crack paths, and consume additional crack-propagation energy, thereby improving damage tolerance in the ceramic matrix [26]. However, introducing high dislocation densities into ceramics remains challenging.

Some UHTCs, particularly high-entropy or multicomponent systems, may undergo local microstructural phase transformations in regions subjected to localized heating or stress concentration. Such transformations can absorb crack-tip energy or generate local compressive stresses that suppress crack propagation [23].

Microstructural designs, such as layered architectures and fibrous monolithic structures, can improve fracture toughness [27]. In many cases, microstructural design is more effective than simply adding toughening phases [23], and the fracture toughness can exceed 10 MPa·m^1/2^ [27].

Extrinsic toughening mechanisms mainly retard crack propagation through the introduction of reinforcing phases, including crack deflection, fiber bridging, fiber pull-out, and crack branching.

The introduction of second-phase particles, whiskers, and fibers can effectively induce crack deflection, bridging, and pull-out, thereby substantially improving damage tolerance [28,29,30,31,32,33,34,35,36]. Continuous fiber reinforcement can also mitigate the poor thermal-shock resistance of UHTCs [30,31,32]. In addition, soft phases such as graphene, nanosheets, and carbon nanotubes (CNTs) can interact with ceramic nanoparticles to suppress crack propagation and thus contribute to strengthening and toughening [35,37]. Microstructural design is often more effective than the simple addition of toughening phases in improving fracture toughness [23]. SiC, BN, ZrO_2_, Mo, MoSi_2_, and graphite with different morphologies and sizes can also markedly affect the properties of the final sintered products [28,29]. SiC is the most widely used oxidation-resistant reinforcement in ZrB_2_/HfB_2_-based UHTCs. At intermediate temperatures, it can oxidize to form SiO_2_ or borosilicate glass, which seals pores, reduces oxygen diffusion, and improves thermal-shock stability. However, above 2000 °C or under low oxygen partial pressure, SiC can undergo active oxidation to generate SiO(g) and CO(g), while the SiO_2_ glass phase may soften, volatilize, or be removed by high-speed flow. Therefore, its long-term protective effect depends on whether it can form a continuous, dense, and erosion-resistant composite oxide scale together with a ZrO_2_/HfO_2_ skeleton [23,28,38]. Carbon-based reinforcements, such as carbon fibers, CNTs, and graphene, can improve toughness and thermal-shock resistance at room temperature or in inert atmospheres through crack deflection, bridging, pull-out, and enhanced thermal conductivity. However, they are intrinsically unstable in oxidizing atmospheres and must be protected during long-term service at 2000 °C by dense UHTC/SiC matrices, pyrolytic carbon (PyC)/SiC interphases, or external oxidation-resistant coatings; otherwise, oxidation of carbon fibers may form connected channels and weaken load-bearing capacity [35]. For CNTs and graphene, Jin et al. showed that CNTs can significantly improve the strength and fracture toughness of HfB_2_, whereas Ni et al. noted that CNTs may react with oxide impurities in UHTCs and deteriorate mechanical properties. Simonenko et al. further found that 1 vol.% graphene in HfB_2_-30 vol.% SiC-graphene can prolong the time during which the surface temperature remains below 1800–1850 °C and reduce the ablation rate in a supersonic dissociated-air jet for 2000 s, but cannot fully prevent the surface temperature from increasing to 2300–2400 °C. This indicates that graphene mainly improves thermal conductivity and delays thermal instability rather than serving as a long-term oxidation-stable phase [23,33,36]. BN platelets can improve the fracture toughness and thermal-shock resistance of ZrB_2_-SiC composites through weak interfaces, crack deflection, and stress relaxation. However, the oxidation product B_2_O_3_ volatilizes readily at high temperature. Ni et al. reported that B_2_O_3_ formed by oxidation of BN interfaces volatilizes strongly above 1100 °C. Therefore, BN is more suitable as an intermediate-temperature weak-interface/toughening phase and should not be regarded as a long-term stable reinforcement in oxidizing environments at 2000 °C [23,29].

In general, extrinsic toughening mechanisms often contribute more directly to composite toughness by delaying crack propagation. Dislocation toughening has limited effectiveness in ceramics, whereas transformation toughening requires strict temperature and stress conditions and may rapidly lose effectiveness at high temperature. Crack bridging, crack deflection, and fiber pull-out are comparatively easier to activate and usually provide stronger toughening effects. Intrinsic mechanisms provide baseline toughness through grain-size control, lattice distortion, and transformation toughening. Combining continuous fiber reinforcement with nanoparticle introduction can simultaneously optimize intrinsic and extrinsic toughening effects and thereby substantially improve toughness [39].

**Figure 2:**
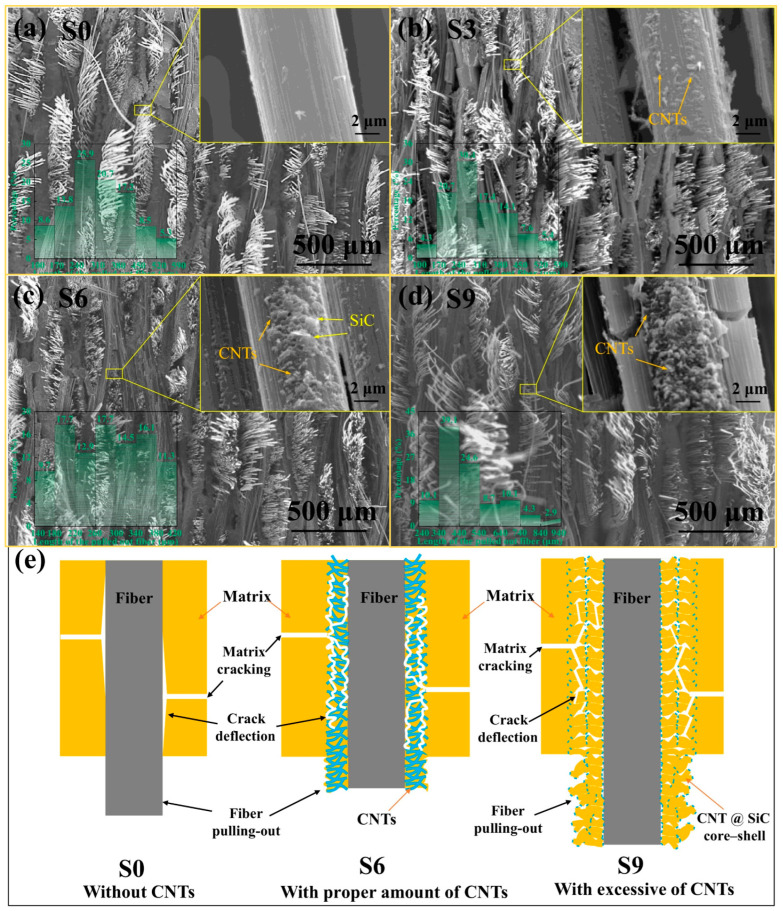
Flexural fracture morphology of Cf/CNTs–PyC/SiC composites: (**a**) S0, (**b**) S3, (**c**) S6, and (**d**) S9. (**e**) Strengthening and toughening mechanisms of Cf/CNTs–PyC/SiC composites. Reproduced from [40] under the terms of the Creative Commons Attribution 4.0 International License (CC BY 4.0).

Figure 2 elucidates the effect of CNT growth duration on the fracture characteristics of C_f_/CNTs–PyC/SiC composites, as well as the underlying reinforcement and toughening mechanisms. In the CNT-free sample (Figure 2a, S0), fracture is primarily dominated by fiber pull-out, exhibiting a bundle-like morphology. With the introduction of an appropriate amount of CNTs (Figure 2b,c, S3 and S6), the fracture morphology evolves into a hybrid mode characterized by brush-like and filamentous fiber pull-out. This transition reflects a substantial enhancement in both flexural strength and toughness. In contrast, excessive CNT growth (Figure 2d, S9) results in the formation of a thick and porous CNT network on the fiber surface. This overgrowth impedes the infiltration of the SiC precursor, leading to reduced matrix densification.

The schematic illustration in Figure 2e further clarifies the evolution of strengthening and toughening mechanisms. For the CNT-free composite (S0), the dominant mechanisms include matrix cracking, crack deflection, interfacial debonding, fiber pull-out, and fiber bridging. Upon incorporation of an optimal CNT content (S6), additional mechanisms such as CNT-induced crack bridging, “nail-like” reinforcement, extended crack deflection, and enhanced pull-out are activated, along with CNT-assisted densification of the matrix. However, in the case of excessive CNTs (S9), the reduced densification of the SiC matrix and the formation of a CNT-rich interphase (core–shell structure) hinder effective stress transfer from the matrix to the fibers, ultimately diminishing both strength and toughness [40]. Excessive CNT incorporation may also introduce undesirable porosity. Therefore, controlling CNT content is essential for fully exploiting its strengthening and toughening effects.

Compared with carbides and nitrides, transition-metal diborides possess an AlB_2_-type hexagonal layered crystal structure and generally exhibit relatively high and anisotropic strength. Diborides with a “core–shell” microstructure, in which solid-solution diboride phases encapsulate pure diboride grain seeds, can achieve strengths as high as 660 MPa at 2100 °C. Such structures exhibit remarkable compressive resistance at ultra-high temperatures and may even enhance oxidation behavior [41]. Diborides typically exhibit relatively high elastic moduli, approximately 480–560 GPa, and moderate hardness values in the range of 20–35 GPa. Among these materials, zirconium diboride (ZrB_2_) has been studied most extensively because of its relatively low density (6.08 g·cm^−3^), high melting point (3250 °C), high thermal conductivity (60–130 W·m^−1^·K^−1^), high electrical conductivity, and good high-temperature strength. ZrB_2_- and HfB_2_-based ultra-high-temperature ceramics are capable of maintaining long-term non-ablative behavior in oxidative environments above 2000 °C, making them highly promising candidates for non-ablative ultra-high-temperature thermal protection materials [27].

The ideal tensile strength of a defect-free diboride crystal can be roughly estimated using a Griffith-type bond-breaking approximation [42]:

(1) $$\sigma_{th}=\sqrt{\frac{E\gamma }{a_{0}}}$$

where $\sigma_{th}$ is the ideal theoretical strength, and E, γ and $a_{0}$ represent the elastic modulus, surface energy, and average interatomic spacing, respectively. Here:

(2) $$\gamma \approx \frac{E\cdot a_{0}}{40}$$

(3) $$\sigma_{th}\approx \frac{E}{\sqrt{40}}$$

This value should be regarded only as an upper-bound estimate for an ideal, defect-free crystal. It should not be confused with the measured flexural strength or fracture strength of real UHTCs, which is usually much lower because of porosity, grain boundaries, residual stresses, microcracks, anisotropic bonding, and processing-induced defects. The incorporation of SiC particles into diborides is a commonly adopted toughening strategy. Finer initial SiC particles can effectively reduce the grain size of ZrB_2_ and HfB_2_ and promote a morphological transformation of the SiC phase from equiaxed grains to whisker-like structures. The resulting ZrB_2_–SiC ceramics exhibit the highest flexural strength under optimized conditions. However, when the SiC particles are excessively fine or added in excessive amounts, the elongated morphology of the SiC second phase may become overly pronounced, which can deteriorate the mechanical properties of the material [27].

Continuous fiber reinforcement can transform the failure mode of the material from instantaneous brittle fracture to progressive damage accumulation. The introduction of fibers activates mechanisms such as crack deflection and energy dissipation, thereby significantly enhancing fracture resistance.

**Figure 3:**
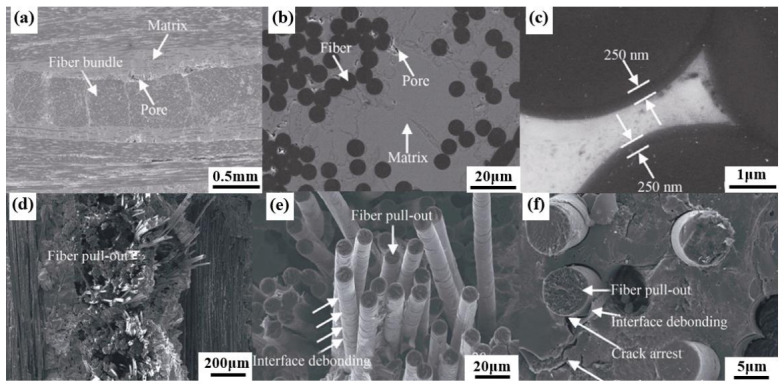
SEM images of Cf/SiBCN composites. Panels (**a**–**c**) show polished cross-sections, and panels (**d**–**f**) show fracture-surface morphologies and toughening features, including crack deflection, interfacial debonding, and fiber pull-out. Adapted from [32] under the terms of the Creative Commons Attribution 4.0 International License (CC BY 4.0).

As shown in Figure 3a–c, carbon fiber–reinforced SiBCN-based composites (Cf/SiBCN) exhibit a highly dense microstructure in which the fibers are tightly bonded with the SiBCN matrix. Figure 3d shows extensive fiber pull-out, while Figure 3f reveals multiple toughening mechanisms within the composite, including crack propagation, crack arrest, interfacial debonding, and short-fiber pull-out. These mechanisms collectively contribute to the high fracture toughness of the composite material [32].

Due to the difference in coefficients of thermal expansion between fibers and ceramic matrices, nonuniform thermal expansion occurs between the fibers and the matrix as temperature increases, which weakens the interfacial bonding between them. As a result, mechanisms such as crack deflection and fiber pull-out become more likely, contributing to an increase in the tensile strength of the composite. However, excessively large differences in thermal expansion coefficients may generate significant residual stresses, which can promote crack initiation. Furthermore, at temperatures above approximately 1800 °C, fiber degradation may occur, leading to a reduction in the mechanical strength of the composite [43].

Although these composites are reinforced with continuous fibers, the matrix regions between fiber bundles and between layers still exhibit brittle behavior at the micrometer scale. To address this limitation, some studies have introduced one-dimensional nanostructures into the matrix—such as CNTs, boron nitride nanotubes (BNNTs), and silicon carbide nanowires (SiCNWs) [44]—to develop hierarchically reinforced composites. The incorporation of PyC and PyC/SiC interphases has also been shown to improve the damage tolerance of ultra-high-temperature ceramic matrix composites (UHTCMCs) [45]. Coating fibers with an appropriately thick PyC interphase can reduce chemical reactions between fibers and oxide impurities, promote mechanisms such as fiber pull-out and crack deflection, and provide a weak interfacial bonding strength that helps release stresses and enhance overall toughness [46].

The incorporation of HfC nanowires as a toughening phase in ZrB_2_–SiC/SiC composites has been shown to improve hardness, elastic modulus, and fracture toughness due to the presence of HfC nanowires. Similarly, the introduction of SiC/PyC nanowires into ZrB_2_–SiC/SiC and TaB_2_–SiC materials [47] has been demonstrated to effectively alleviate thermal-stress concentration and enhance ceramic toughness. Among nanoscale reinforcements, ceramic whiskers and nanowires are the most widely utilized.

The toughening mechanisms associated with nanoscale reinforcements can generally be summarized into three main aspects. First, nanoscale reinforcing phases can induce crack deflection at the crack tip, thereby reducing the stress intensity factor and hindering crack propagation. Second, the pull-out of reinforcing phases generates new fracture surfaces, which consumes additional energy during fracture. Third, the reinforcing phases can bridge cracks, providing resistance against crack propagation.

In addition to reinforcement phases, a high density of dislocations can induce localized plasticity at ceramic crystal surfaces. The stress fields surrounding dislocations may also enhance fracture toughness through a stress-shielding effect [26]. However, introducing a high density of dislocations into ceramic materials remains challenging.

Overall, monolithic UHTCs possess excellent hardness, stiffness, and thermal stability but suffer from limited fracture toughness. Therefore, the current research generally adopts composite design to achieve toughening and reliability improvement. In addition, computational design methods can improve the efficiency of identifying materials with desirable properties in large compositional spaces. For example, the bonding strength between different materials can be explored by using simulations such as first-principles calculations to screen better mechanical properties [48].

### 3.2. Thermal Conductivity

Thermal conductivity is a critical parameter for UHTCs used in thermal protection systems because it directly affects local heat dissipation, temperature-gradient formation, and thermal-stress accumulation. Unlike conventional oxide thermal-barrier materials, transition-metal carbides, nitrides, and diborides generally exhibit mixed metallic and covalent bonding. Therefore, their total thermal conductivity can be considered as the combined contribution of electronic and phononic heat transport, and is commonly expressed as:

(4) $$\kappa_{\mathrm{total}}=\kappa_{e}+\kappa_{\mathrm{ph}}$$

where $\kappa_{e}$ and $\kappa_{\mathrm{ph}}$ are the electronic and phononic thermal conductivity, respectively. The electronic thermal conductivity can be estimated using the Wiedemann–Franz relation [49]:

(5) $$\kappa_{e}=L\sigma T$$

where L is the Lorenz constant, σ is the electrical conductivity, and T is the absolute temperature. The phonon thermal conductivity can be further described quantitatively using the phonon gas model [11]:

(6) $$\kappa_{\mathrm{ph}}=\frac{1}{3}C_{v}v_{g}^{2}\tau$$

where $C_{v}$ is the phonon heat capacity, $v_{g}$ is the phonon group velocity, and τ is the phonon relaxation time. This relationship indicates that any factor that decreases the phonon group velocity or shortens the relaxation time will reduce the lattice thermal conductivity. Local disorder can enhance Rayleigh-type phonon scattering, shorten the phonon mean free path, and thereby lower lattice thermal conductivity. To quantitatively analyze the effects of lattice distortion, vacancies, grain boundaries, and anharmonicity on thermal conductivity, the phonon relaxation time can be expressed as the combined contribution of multiple scattering processes. According to Matthiessen’s rule, the total phonon scattering rate can be written as:

(7) $$\tau_{\mathrm{total}}^{-1}=\tau_{U}^{-1}+\tau_{B}^{-1}+\tau_{I}^{-1}+\tau_{V}^{-1}+\tau_{e\text{-}\mathrm{ph}}^{-1}$$

where the $\tau_{U}^{-1}$, $\tau_{B}^{-1}$, $\tau_{I}^{-1}$, $\tau_{V}^{-1},\;\mathrm{and}\;\tau_{e\text{-}\mathrm{ph}}^{-1}$ represent Umklapp, grain-boundary, mass fluctuation, vacancy, and electron–phonon scattering relaxation times, respectively. In HE-UHTCs, random occupation by multiple principal elements significantly enhances mass fluctuation scattering. Lattice distortion and local bond-strength variations increase force-constant-disorder scattering, whereas nanocrystals, second phases, and interfaces enhance boundary scattering. The mass fluctuation and lattice distortion induced by high-entropy design can be semi-quantitatively described using point-defect scattering parameters. Mass fluctuation scattering is generally related to the squared deviation of each constituent mass from the average mass and can be expressed as:

(8) $$\Gamma_{\mathrm{mass}}=\underset{i}{\sum }c_{i}{\left(1-\frac{m_{i}}{\bar{m}}\right)}^{2}$$

where $c_{i}$ and $m_{i}$ are the atomic fraction and atomic mass of the i-th component, respectively, and $\bar{m}$ is the average atomic mass. For HE-UHTCs, strain-field scattering caused by atomic-radius mismatch should also be considered and can be expressed analogously as:

(9) $$\Gamma_{\mathrm{strain}}=\underset{i}{\sum }c_{i}{\left(1-\frac{r_{i}}{\bar{r}}\right)}^{2}$$

where $r_{i}$ and $\bar{r}$ are the atomic radius of the i-th component and the average atomic radius, respectively. Therefore, when multiple metallic elements such as Hf, Zr, Ti, Ta, Nb, Mo, and W share the cation sublattice, both mass fluctuation scattering and strain-field scattering are introduced. In addition, differences in metal–nonmetal bond strength generate force-constant disorder, which modifies the phonon dispersion relationship and phonon group velocity. These effects collectively reduce the phonon relaxation time and mean free path and are key mechanisms responsible for the reduced lattice thermal conductivity of HE-UHTCs. Accordingly, the low lattice thermal conductivity of high-entropy ceramics can be quantitatively understood as the result of a reduced phonon mean free path caused by multiple superimposed scattering channels [11,50,51].

In UHTCs, strong covalent bonding generally favors high phonon group velocity; therefore, diborides such as ZrB_2_ and HfB_2_ exhibit relatively high thermal conductivity. In HE-UHTCs, however, mass fluctuation, atomic-size mismatch, local strain, and bond-strength variation caused by multicomponent solid solution increase phonon scattering frequency and shorten phonon relaxation time, thereby reducing phonon thermal conductivity. Thus, the primary effect of high-entropy design on thermal conductivity is not simply that more elements necessarily produce lower thermal conductivity; rather, it regulates thermal transport by modifying phonon group velocity and phonon relaxation time [52].

For diborides such as ZrB_2_ and HfB_2_, atomistic simulations have shown that the total thermal conductivity of these high-thermal-conductivity UHTCs contains both electronic and phononic carrier contributions. The electronic contribution can be estimated from electrical conductivity using the Wiedemann-Franz relation, whereas the phononic contribution can be directly analyzed using MD or the Green-Kubo method [49]. For carbides such as ZrC, first-principles calculations also indicate the coexistence of electron-phonon interactions and phonon-phonon scattering. The electronic contribution becomes more important at elevated temperature, whereas the phonon thermal conductivity is mainly controlled by phonon-phonon scattering, defect scattering, grain-boundary scattering, and vacancy scattering [50].

The relative contributions of phononic and electronic thermal transport vary among different UHTC systems. Diborides such as ZrB_2_ and HfB_2_ possess an AlB_2_-type layered structure. The strong B-B covalent network favors high phonon velocity, while the transition-metal layers provide a certain degree of metallicity. As a result, diborides usually exhibit high total thermal conductivity and pronounced anisotropic thermal transport [49]. Carbides such as ZrC, HfC, and TaC have rock-salt structures and mixed metallic-covalent bonding, so electronic thermal conduction is non-negligible. Carbon vacancies, grain size, and impurities can also substantially change phonon scattering intensity. Nitrides such as ZrN, HfN, and TiN also exhibit partial metallicity, but their thermal conductivities are strongly influenced by nitrogen vacancies, nonstoichiometry, and changes in electronic structure [23]. Therefore, the thermal conductivity of UHTCs should not be empirically ranked only by whether they are carbides, nitrides, or borides; instead, crystal structure, bonding characteristics, electronic density of states, defect concentration, grain size, and testing temperature should be considered together.

For HE-UHTCs, co-occupation of the cation sublattice by multiple transition-metal elements such as Hf, Zr, Ti, Ta, Nb, Mo, and W further introduces mass fluctuation, size mismatch, bond-strength variation, and local strain fields, thereby significantly enhancing phonon scattering. Transition-metal carbides, nitrides, and borides generally possess mixed metallic and covalent bonding, and some UHTCs exhibit pronounced metallic character. Therefore, the electronic thermal contribution cannot be ignored. Meanwhile, strong M–C, M–N, or B–B covalent networks affect phonon velocity, phonon scattering, and lattice thermal conductivity. Thus, the thermal conductivity of HE-UHTCs should be regarded as the combined result of electronic thermal conduction, lattice thermal conduction, and microstructural thermal resistance [53]. Table 1 presents the mechanical and thermal performance diagrams related to ultra-high temperature and room temperature.

### 3.3. Thermal Stability and Thermal Shock Resistance

Crystal-structure anisotropy can strongly influence the thermal-shock behavior of UHTCs. FCC carbides and nitrides are relatively isotropic crystallographically, and their thermal expansion and thermal conduction are less direction-dependent. In contrast, AlB_2_-type hexagonal diborides have layered structures, and their thermal conductivity, coefficient of thermal expansion, and elastic modulus may differ significantly along the a- and c-axes. During thermal shock, this anisotropy can lead to orientation-dependent thermal-stress concentration, grain-boundary mismatch, and microcrack initiation [2,54].

Due to their intrinsic brittleness, UHTCs are susceptible to thermal shock damage under extreme temperature gradients. Two parameters are commonly used to evaluate thermal shock resistance: the thermal shock damage parameter (R) and the thermal shock fracture parameter (R′′′′) [27]:

(10) $$R=\frac{\sigma_{f}\left(1-\upsilon \right)}{E\alpha }$$

(11) $$R^{\prime \prime \prime \prime }=\frac{E\gamma_{s}}{\sigma_{f}^{2}\left(1-\upsilon \right)}=\frac{K_{\mathrm{IC}}^{2}}{2\sigma_{f}^{2}\left(1-\upsilon \right)}$$

where $\sigma_{f}$ is the fracture strength of the material, υ is Poisson’s ratio, E is the elastic modulus, α is the coefficient of thermal expansion, $\gamma_{s}$ is the fracture surface energy, and $K_{\mathrm{IC}}$ is the fracture toughness.

The thermal shock damage parameter reflects the ability of a material to resist cumulative damage during repeated thermal cycling, whereas the thermal shock fracture parameter is primarily related to fracture toughness and strength. As indicated by Equation (10), when the elastic modulus, coefficient of thermal expansion, and Poisson’s ratio remain constant, improving the strength of UHTC composites is beneficial for enhancing their resistance to thermal shock.

However, large differences in thermal expansion coefficients between the matrix and reinforcement phases may introduce substantial residual stresses during processing. For example, in ZrB_2_- or HfB_2_-based composites containing SiC, thermal mismatch during high-temperature sintering can generate internal stresses that reduce thermal shock resistance. Therefore, reducing thermal stress must be considered in materials design. For example, the incorporation of high-melting-point soft phases such as carbon fibers or graphite can help release residual stresses generated during processing, thereby improving the thermal shock resistance of UHTCs.

Microstructural design strategies, including layered architectures and fiber-monolith structures, have also proven effective in enhancing thermal shock resistance. For instance, fiber-monolith ZrB_2_-based UHTCs can achieve a critical thermal shock temperature difference as high as 1400 °C, representing a 250% improvement compared with conventional ZrB_2_ materials [55]. As indicated by Equation (11), the thermal shock fracture parameter is proportional to $K_{\mathrm{IC}}^{2}$/$\sigma_{f}^{2}$; therefore, improving the ratio $K_{\mathrm{IC}}$/$\sigma_{f}$ is essential for enhancing thermal shock resistance. The addition of graphite can significantly increase the ratio between fracture toughness and strength, leading to more than a twofold improvement in the thermal shock fracture parameter [56].

SiC-reinforced ceramics, when exposed to high-enthalpy and high-heat-flux aerodynamic heating environments, may experience volatilization of SiO_2_ formed during oxidation. This process alters the catalytic radiation characteristics of the oxide layer surface, causing sudden fluctuations in surface temperature [57]. Such conditions impose more stringent requirements on the thermal shock resistance of UHTCs.

Functionally graded design of UHTCs provides an effective strategy for addressing these challenges. Researchers have developed layered structures by combining high-temperature materials with similar thermal expansion coefficients. The enhanced performance of such systems can be attributed to stress relaxation and crack deflection within the multilayer architecture [58]. This approach not only reduces stresses generated by mismatches in thermal expansion between coatings and substrates but also enables the coating to better retain its intrinsic protective properties. For example, a two-step SiC coating can be employed, where the inner layer consists of b-SiC, and the outer layer is composed of a-SiC, Si, and b-SiC [59]. In addition to improving fracture toughness [60], graded structural designs can also enhance ablation resistance [61].

**Table 1:** Basic physical, mechanical, and oxidative properties of various ultra-high-temperature ceramics.

Material	Crystal Structure	Processing Conditions (°C/min/MPa)	Melting Point (°C)	Relative Density (%)	CTE (α; 10^−6^ K^−1^)	Thermal Conductivity (W·m^−1^·K^−1^)	Electrical Resistivity (µΩ·cm)	Elastic Modulus (GPa)	Vickers Hardness (GPa)	Fracture Toughness (MPa·m^1/2^)	Flexural Strength (MPa)	Refs.
Carbides												
HfC	FCC	—	3900	—	6.3	22.2	45	461	24.2	—	—	[23,62]
TaC	FCC	SPS, 2300/—/30	3800	92	6.6–8.4	22.2	30–42.1	537	17	—	—	[23,54,62]
ZrC	FCC	HPHT, —/—/—	3530	98.5	6.82	20.61	68	387	25	—	—	[23,54,62]
TiC	FCC	SPS, 1600/10/50	3067	96.1	7.5–7.7	17–21	52.5	437	23.6	4	240—270	[23,62]
Nitrides												
TiN	FCC	—	2950	—	9.35	29.1	21.7	400	18.6	—	—	[23,62]
ZrN	FCC	SPS, 2100/10/50	2950	97.7	7.24	20.9	13.6	384	15	—	—	[23,62,63]
HfN	FCC	—	3385	—	6.5	21.6	33	398	16.1	—	—	[23,62]
TaN	FCC	—	2900	—	3.2	8.3	128–135	490	10.8	—	—	[23,62]
Borides												
TiB_2_	Hexagonal	PS, 1800–2275/60/—	3225	99	8.6	60–120	10–30	500–560	25–35	5–7	71–325	[64,65]
ZrB_2_	Hexagonal	HP, 2100/60/32	3245	97	—	—	—	346	13.7	3.1	392	[66,67]
HfB_2_	Hexagonal	HP, 1800/10/30	—	<89.5	—	—	—	—	18	3.1	259	[68]
TaB_2_	Hexagonal	HPHT, 1400/20/5500	3200	95.11	—	—	—	402	26	—	—	[69,70]

Notes: Processing conditions are expressed as sintering temperature (°C)/holding time (min)/applied pressure (MPa); Abbreviations for processing methods: SPS: Spark Plasma Sintering; HP: Hot Pressing; HPHT: High-Pressure–High-Temperature sintering; PS: Pressureless Sintering. Properties listed correspond to dense, bulk specimens; values may vary depending on starting powder purity, particle size, and sintering additive content.

## 4. High-Temperature Oxidation and Ablation Behavior

### 4.1. Oxidation Mechanisms of UHTCs

Under high-temperature oxidative environments, UHTCs typically form oxide products such as Ta_2_O_5_, HfO_2_, ZrO_2_, and B_2_O_3_. The type of oxide formed, its density, and its volatilization behavior vary significantly among different UHTC systems during oxidation. Among high-temperature carbides, TaC and HfC have been studied most extensively. TaC begins to oxidize at approximately 400 °C and becomes fully oxidized at around 850 °C, forming Ta_2_O_5_, which has a melting point of 1872 °C [71].

Similarly, the oxidation of HfC and ZrC leads to the formation of monoclinic HfO_2_ and ZrO_2_, respectively. Both compounds are stable refractory oxides with melting points of approximately 2800 °C and 2700 °C. In particular, ZrO_2_ exhibits an extremely low vapor pressure and remains highly stable at elevated temperatures [72]. In contrast, B_2_O_3_ possesses a relatively high vapor pressure and can undergo significant volatilization at high temperatures.

ZrB_2_ provides better oxidation protection below about 1200 °C because its oxidation produces a ZrO_2_ layer containing molten boron oxide [73]. The liquid B_2_O_3_ phase can fill cracks and pores, thereby improving the integrity of the protective oxide scale. However, as the temperature increases further, the rapid volatilization of B_2_O_3_ significantly reduces its protective effectiveness.

Kuriakose and Margrave first reported the oxidation kinetics of ZrB_2_ [74], demonstrating that the oxidation of ZrB_2_ in the temperature range of 945–1256 °C under 1 atm O_2_ follows a parabolic rate law. At 1056 °C, the rate constant was found to be proportional to the oxygen partial pressure, indicating that oxygen diffusion controls the oxidation rate. Oxygen diffusion is primarily controlled by transport through the oxide scale. As oxidation time and temperature increase, cracking or porosity may develop in the oxide scale, allowing oxygen to rapidly penetrate into the substrate. In addition, oxygen vacancies can provide fast diffusion pathways, increase the oxygen diffusion rate in the oxide layer, and alter the local chemical potential, resulting in nonuniform oxide-scale growth. The oxidation behavior is governed by the combined effects of solid oxide phases, liquid glass, pores, and the monoclinic-to-tetragonal transformation of oxide phases.

The oxidation rate of ZrB_2_ increases sharply at approximately 1127 °C, while a similar increase occurs for HfB_2_ at about 1627 °C [75].

Between 800 and 1400 °C, the evaporation rate of B_2_O_3_ follows a linear relationship with temperature. Tripp and Graham proposed a quasi-linear equation to describe the oxidation process of ZrB_2_ [76], expressed as:

(12) $$\frac{{\Delta \mathrm{nO}}_{2}}{A}=at^{1/2}+bt$$

At temperatures below 1100 °C, the parabolic term $at^{1/2}$ predominates, indicating that the reaction kinetics are governed by the diffusion of oxygen through the liquid B_2_O_3_ layer. When the temperature exceeds 1400 °C, the linear term becomes dominant due to the volatilization of the protective B_2_O_3_(l) layer. The substantial volatilization of B_2_O_3_(l) at elevated temperatures compromises the oxidation resistance of the material.

The oxidation process of ultra-high-temperature carbide ceramics involves multiple physicochemical steps, including the inward diffusion of oxygen, the formation of oxide phases, and the release of gaseous products such as carbon monoxide and carbon dioxide. This process inevitably introduces structural defects, including pores, thermal stresses, and internal stresses arising from microstructural evolution. These phenomena lead to the initiation of microcracks and progressive surface spallation. From a mechanistic perspective, the oxidation behavior of various types of ultra-high-temperature ceramics (UHTCs) exhibits fundamental differences at elevated temperatures. During the oxidation of carbides and nitrides, the nonmetallic elements (C or N) escape in the form of gaseous species such as CO, CO_2_, or N_2_, which tend to generate porosity within the oxide scale, thereby compromising its density and protective capability. In contrast, diborides can form liquid B_2_O_3_ in the intermediate- to high-temperature range, which binds oxide particles and improves oxide-scale integrity. Within a certain temperature window, B_2_O_3_ has a relatively low vapor pressure and can act as an effective oxygen-diffusion barrier, thereby significantly delaying further oxidation of the substrate. However, when the temperature exceeds approximately 1200–1800 °C, or under high-velocity aerodynamic flow conditions, the rapid volatilization of B_2_O_3_ leads to the gradual degradation of its protective function. Although certain refractory oxides may offer transient protection over short time scales, their inherent volatility and tendency to form porous structures often result in the rapid loss of oxidation resistance.

At the ceramic-oxide interface, the diffusion of oxygen occurs through pores within the oxide layer. The oxygen concentration is governed by its partial pressure, which in turn depends on the temperature. Variations in temperature further influence the diffusion coefficient [77]. Assuming that the cross-sectional area of the oxide layer perpendicular to its growth direction (i.e., the direction along which the thickness L is measured) remains equivalent to that of the parent material and does not change over time, the evolution of oxide thickness L as a function of time t is determined by the porosity f of the oxide scale [78]:

(13) $$\begin{array}{c} \frac{dL}{dt}=\left(\frac{1}{1-f}\right)\left(\frac{1}{\rho_{{\mathrm{HfO}}_{2}}}\right)\frac{dW_{{\mathrm{HfO}}_{2}}}{dt}\\ =\left(\frac{1}{1-f}\right)\frac{2}{5}J_{O_{2}}\frac{M_{{\mathrm{HfO}}_{2}}}{\rho_{{\mathrm{HfO}}_{2}}}, \end{array}$$

where $\rho_{{\mathrm{HfO}}_{2}}$, $W_{{\mathrm{HfO}}_{2}}$, $M_{{\mathrm{HfO}}_{2}}$ and $J_{O_{2}}$ represent the density, mass, molar mass of the oxide, and the oxygen flux, respectively. A higher oxide porosity f facilitates oxidation by allowing easier oxygen transport through the oxide layer. The gas diffusion regime can be evaluated using the Knudsen number:

(14) $$Kn=\lambda_{g}/r_{p}$$

where $\lambda_{g}$ is the gas mean free path and $r_{p}$ is the pore radius. When Kn≪1, intermolecular gas collisions dominate and transport is mainly described by binary molecular diffusion, for which the diffusion coefficient $D_{1-2}$ should be used. When Kn≫1, collisions between gas molecules and pore walls dominate and transport follows Knudsen diffusion, for which $D_{K}$ should be used. For the transition pore-size regime commonly observed in high-temperature oxide scales, namely Kn approximately 1, both mechanisms jointly limit gas transport, and the Bosanquet relation can be used to describe the combined pore diffusion coefficient:

(15) $$D_{K}=\frac{4}{3}{\left(\frac{8RT}{\pi M}\right)}^{1/2}K_{0}$$

(16) $$\frac{1}{D_{eff}}=\frac{1}{D_{K}}+\frac{1}{D_{1-2}}$$

After further considering the oxide scale porosity and pore tortuosity, the effective diffusion coefficient can be written as:

(17) $$D_{\mathrm{eff}}=\frac{f}{\tau }D_{\mathrm{pore}}=\frac{f}{\tau }{\left(\frac{1}{D_{K}}+\frac{1}{D_{1-2}}\right)}^{-1}$$

where f is the open porosity, τ is the pore tortuosity, $D_{\mathrm{pore}}$ is the combined gas diffusion coefficient within the pores, and $D_{1-2}$ is the binary molecular diffusion coefficient between oxygen and the other gas species. This expression indicates that the effective mass-transport capability of the oxide scale depends not only on porosity but also on pore size, pore connectivity, tortuosity, and the relative contributions of molecule–wall and molecule–molecule collisions. Therefore, dense oxide scales with low pore connectivity and high tortuosity are more effective in reducing oxygen diffusion flux, whereas porous, cracked, or highly connected pore networks substantially weaken the protective effect [78].

Therefore, the compactness of the oxide layer—rather than merely the type of oxide formed—is often the key factor determining the oxidation resistance of UHTCs. This understanding provides a theoretical basis for improving oxidation resistance through microstructural control and composite design.

Introducing oxidation-resistant additives such as SiC is an effective strategy for improving oxidation resistance. During oxidation, these additives promote the formation of a high-viscosity borosilicate glass layer. According to the Stokes–Einstein relationship, an increase in viscosity may reduce the diffusion rate of oxygen:

(18) $$D_{liq}=\frac{kT}{3\pi \lambda \eta }$$

where k is the Boltzmann constant, T is the thermodynamic temperature, λ represents the hydrated ionic diameter, and η is the viscosity. The liquid-phase diffusion coefficient $D_{\mathrm{liq}}$ is inversely proportional to the viscosity η.

At present, most oxidation-resistance strategies rely on introducing multiple oxidation-resistant additives to promote the formation of stable and dense oxide layers on the material surface. Common oxidation-resistant additives include silicon carbide, oxides [79], and silicides. Grain refinement can improve the uniform distribution of these additives, effectively filling voids and bridging defects, thereby inhibiting oxygen ingress and enhancing oxidation resistance. However, excessively fine grain sizes may adversely affect the high-temperature mechanical properties of the material.

**Figure 4:**
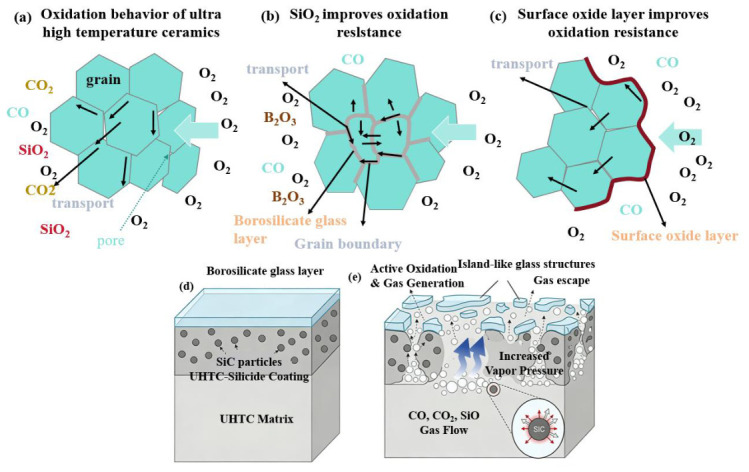
Schematic illustration of oxygen transport and oxidation-protection mechanisms in UHTC matrices: (**a**) without additives, (**b**) with SiC addition, (**c**) with a surface antioxidant layer, and (**d**,**e**) island-like coating morphologies after ablation [81].

Among various additives, the incorporation of SiC has proven particularly effective in improving the oxidation and ablation resistance, as well as the overall performance, of UHTCs. As illustrated in Figure 4, oxidation of SiC generates SiO_2_, which can cover the material surface or fill the pores within the ZrO_2_ skeleton structure, thereby providing effective oxidation protection [72]. However, at temperatures above approximately 1850 °C, SiO_2_ may lose its protective capability due to rapid volatilization. To address this limitation, researchers have attempted to introduce rare-earth oxides and TiC into the matrix to stabilize the oxidation products and increase the melting point of silica, thereby further enhancing the ablation resistance of UHTCs [80].

During ablation testing, UHTCs are subjected to even more severe aerodynamic loads and higher temperatures. Under such extreme conditions, active oxidation of SiC generates a larger amount of gaseous by-products. Owing to the increased vapor pressure, these gaseous species may penetrate the top glassy layer, leading to the formation of an “island-like” morphology on the surface of UHTC–silicide coatings and thereby disrupting the continuity of the protective glass layer [82], as shown in Figure 4d,e. When the ablation temperature exceeds 2000 °C, the ablation resistance of ceramics depends predominantly on transition-metal (TM) oxides, such as ZrO_2_ and HfO_2_. However, volume changes associated with phase transformations in TM oxides can compromise the integrity of the oxide layer and even cause spallation. The addition of rare-earth (RE) compounds can promote reactions with TM oxides and facilitate the formation of complex oxides such as La_2_Zr_2_O_7_ [83]. Additives such as TaSi_2_ and La_2_O_3_ have also been shown to improve the oxidation resistance of ZrB_2_- and HfB_2_-based ceramic matrix composites [84].

In addition, highly textured MB_2_-based UHTCs have been developed through strong magnetic field alignment. Owing to the intrinsic anisotropy of diborides, these materials exhibit direction-dependent oxidation resistance, with surfaces oriented perpendicular to the c-axis of MB_2_ showing superior oxidation resistance [42].

The oxidation and ablation resistance of UHTCMCs depends to a large extent on the composition and microstructure of the surface oxide layer formed during exposure. Due to the presence of reinforcing fibers, these composites display anisotropic oxidation behavior. Carbon fibers are particularly vulnerable when exposed to oxidative environments and can be rapidly consumed. When oxidation propagates along the fiber layers, the fibers are depleted, leaving pores that facilitate further oxygen diffusion [23]. By contrast, when oxidation proceeds perpendicular to the fiber layers, the fibers are oxidized rapidly and the ceramic matrix becomes exposed. The ceramic matrix can then oxidize to form a denser oxide layer, which provides improved resistance to further oxidation [85].

Simulation of the oxidation behavior of high-temperature ceramics represents a viable and increasingly important research approach. For example, modeling the growth and thickness evolution of oxide layers can provide insights into fracture behavior. In parallel, high-throughput experiments combined with data-driven strategies and machine learning have been employed to predict the oxidation behavior of ceramics [86].

### 4.2. Ablation Behavior

**Figure 5:**
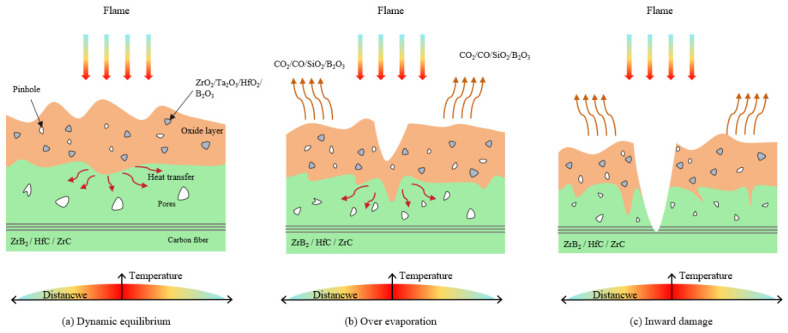
Schematic diagram of ultra-high-temperature ceramic ablation.

Under extreme aerothermal conditions, UHTCs experience mass loss through oxide volatilization, surface melting, and aerodynamic erosion. These processes often act synergistically, resulting in highly complex ablation behavior that cannot be fully captured by oxidation kinetics alone. Under moderate heat flux, SiC oxidation can generate SiO_2_ or borosilicate glass phases that fill pores and cracks and reduce oxygen diffusion. ZrO_2_, HfO_2_, TiO_2_, and their complex oxides can improve the erosion resistance of the oxide layer. In contrast, under high heat flux and high-speed flow, loss of volatile species such as SiO_2_, B_2_O_3_, and SiO intensifies, the viscosity of the glassy phase decreases, and active oxidation of SiC produces SiO(g), CO(g), and CO_2_(g), which can generate pores, bubbles, and local internal pressure, further disrupting the continuity of the oxide scale. In this regime, the ablation mechanism gradually shifts from oxygen diffusion control to a process dominated by volatilization loss, mechanical erosion, and oxide scale spallation. Studies by Pan et al. and Zeng et al. on ZrC-SiC-TiC systems show that high viscosity, low volatility, and a complex-oxide skeleton in multiphase oxide layers help resist high-temperature gas flow erosion, whereas single SiO_2_ or low viscosity oxide layers are more prone to volatilization and spallation above 2000 °C [87,88]. Figure 5 shows three common processes during the ablation stage.

Oxide-scale spallation is also controlled by thermal stress and interfacial structure. Rapid heating and cooling, oxide phase transformations, and thermal expansion mismatch between coatings and substrates can induce cracking and interfacial debonding. Once cracks form, aerodynamic shear can further peel the oxide layer along cracks and pores. Appropriate coating thickness, embedded interfaces, HfO_2_-ZrO_2_ solid-solution sintering, and complex oxide scales such as La_2_Zr_2_O_7_ and Hf-Ta-Si-O can improve spallation resistance by strengthening interfacial bonding, relieving thermal stress, stabilizing ZrO_2_ phase transformations, and increasing oxide-scale viscosity [80,89,90,91,92].

Severe oxidation and structural degradation are the two principal causes of ablation, as they critically influence the ablation tolerance of the material [93]. Consequently, incorporating oxidation-resistant elements such as Ti, Cr, and Si into the material matrix has been widely regarded as an effective strategy for controlling oxidation. Such additions promote the formation of viscous SiO_2_ glass phases and solid solutions such as (Zr,Ti)O_2_ or (Hf,Ti)O_2_, which help seal oxygen diffusion pathways [94]. Alternatively, the addition of high-melting-point oxides such as HfO_2_, LaB_6_, La_2_O_3_, and Y_2_O_3_ can stabilize the ZrO_2_ phase and thereby enhance ablation resistance under extreme ultra-high-temperature conditions.

In addition, the sluggish diffusion effect induced by the high configurational entropy in high-entropy ceramics (HECs) can significantly mitigate oxidation processes in high-temperature environments [95]. Therefore, implementing high-entropy design on both anion and cation sublattices can substantially enhance ablation resistance. Furthermore, some studies have reported that introducing medium-entropy oxides can reduce ablation damage caused by oxidation-induced porosity and thermal shock cracking [96].

In general, the ablation resistance of UHTCMCs is achieved through the formation of high-melting-point oxide layers together with glassy phases that fill the pores within the oxide scale [97]. In multiphase ceramics, the incorporation of SiO_2_ can promote the self-healing of cracks and pores generated during ablation. However, due to the volatility of SiO_2_, this self-healing capability becomes ineffective at temperatures above approximately 1600 °C [98]. At excessively high temperatures, certain oxide glass phases may volatilize, which can reduce the heat load on the material but also weaken the protective capability of the oxide layer against ablation [99]. Meanwhile, the addition of TiC into UHTCMCs can lead to the formation of high-viscosity and low-volatility Zr_1−x_Ti_x_O_2_, which provides improved resistance to high-temperature erosion. The precipitation of Zr-rich oxides can further increase the viscosity of the oxide layer, enabling it to better withstand high-temperature gas flow erosion. Generally, Hf-based UHTCMCs exhibit superior ablation resistance compared with Zr-based composites because the melting point of HfO_2_ is higher than that of ZrO_2_. Moreover, HfB_2_ demonstrates better ablation resistance than HfC because its oxidation products tend to adhere to the composite surface [100].

In addition to composition, thermal properties also exert a significant influence on the ablation behavior of composites. Composites with higher thermal conductivity can more effectively dissipate heat, thereby reducing the surface temperature during ablation and improving ablation resistance [101]. In general, heat is primarily conducted through phonon transport within the solid framework of the composite. Phonons are scattered by pores, grain boundaries, and phase interfaces, which reduces the overall thermal conductivity of the composite. Therefore, reducing porosity and establishing a dense, continuous phase distribution are effective strategies for improving thermal conductivity and consequently enhancing ablation resistance [23]. In addition, carbon fibers are particularly vulnerable during ablation, as they are easily oxidized and may form pathways for oxygen diffusion. As a result, ablation resistance can be improved by optimizing the structure of fiber preforms [38] or by developing ultra-high-thermal-conductivity carbon-fiber-reinforced composites [102].

Furthermore, the effects of ablation can be mitigated through the development of oxidation-resistant coatings, multilayer gradient designs, and controlled oxidation–ablation sequences. For example, thermodynamic diagrams can be used to determine the preferential oxidation sequence of different components at ablation temperatures, allowing the ablation performance of multicomponent UHTCs to be evaluated in advance based on different metal compositions or compositional ratios. By carefully designing the composition and controlling the oxidation sequence with temperature, the ablation resistance of UHTCs can be further improved [103].

It should be noted that laboratory ablation results cannot be directly extrapolated to real hypersonic service environments. Oxyacetylene, laser, and plasma ablation tests represent reactive flames, high thermal shock, and high-enthalpy gas flows, respectively, but they cannot simultaneously reproduce the high-Mach-number boundary-layer shear, shock-wave/boundary-layer interactions, atomic oxygen, pressure gradients, component curvature, and nonuniform heat flux encountered in flight. Future evaluations should therefore report heat flux, surface temperature, gas velocity/pressure, oxygen partial pressure, specimen geometry, exposure time, oxide-scale thickness, spallation area, cross-sectional microstructure, and residual mechanical properties, rather than relying solely on mass or linear ablation rates [75].

## 5. HE-UHTCs

The concept of high-entropy ceramics originated from the development of high-entropy alloys. Unlike traditional single-component or binary systems, HE-UHTCs typically consist of five or more elements occupying equivalent lattice sites in near-equiatomic proportions. The introduction of high configurational entropy fundamentally alters the structure–property relationships of HE-UHTCs. In contrast to conventional UHTCs, whose properties are largely determined by average bonding strength, HE-UHTCs exhibit unique behaviors arising from the combined effects of entropy stabilization, lattice distortion, and chemical disorder. These synergistic effects endow HE-UHTCs with excellent high-temperature oxidation resistance, improved sinterability, strong phase stability, enhanced toughness, and superior ablation resistance [104].

Importantly, the formation of high-entropy ceramics strongly depends on the balance among configurational entropy, mixing enthalpy, atomic size compatibility, and synthesis conditions. Therefore, understanding the thermodynamic principles governing phase stability remains a prerequisite for the rational design of HE-UHTCs.

HE-UHTCs are typically fabricated using techniques such as pressureless sintering (PLS), hot pressing (HP), spark plasma sintering (SPS), and reactive spark plasma sintering (RSPS). Among these methods, HP has been widely employed because it can produce dense HE-UHTCs, although the sintering temperature is often higher than 1800 °C. Owing to its time efficiency, SPS has become the most commonly used technique for densifying high-entropy UHTCs. Most HE-UHTCs are synthesized from the corresponding oxides through borothermal or carbothermal reduction processes [105].

### 5.1. Crystal Structures of HE-UHTCs

Typical crystal structures of high-entropy oxide ceramics include rock-salt, fluorite, perovskite, pyrochlore, and spinel structures. High-entropy oxides (HEOs) with fluorite structures exhibit low thermal conductivity, high thermal stability, and tunable coefficients of thermal expansion. Perovskite-structured HEOs possess good structural stability [106], whereas pyrochlore-structured HEOs are characterized by high elastic moduli [107] and low thermal conductivity [108]. In addition, monoclinic, rutile, and CaF_2_-type structures are also commonly found in HEO systems. Table 2 shows the common high entropy ceramic crystal structures.

The synergistic interactions between different types of anions and cations within ceramic lattices endow these materials with high structural stability and favorable thermal expansion characteristics [109], making them attractive candidates for thermal barrier coatings on the surfaces of turbines, engines, and related high-temperature components. High-entropy nitride ceramics generally adopt a NaCl-type crystal structure and exhibit excellent high-temperature oxidation resistance and hardness, which make them suitable for wear-resistant coatings on high-speed cutting tools and corrosion-resistant coatings on gas-turbine compressor blades. High-entropy borides, owing to their high melting temperatures and excellent thermochemical stability, are regarded as promising ultra-high-temperature ceramics. As structural materials, they are capable of operating under ultra-high-temperature conditions [110], although their thermodynamic performance and stability still require further improvement under extreme service environments. High-entropy diborides possess a distinctive layered hexagonal crystal structure composed of alternating rigid two-dimensional boron networks and high-entropy two-dimensional metal-cation layers, in which mixed ionic and covalent bonding exists between the metal and boron atoms (M–B bonds) [111].

**Table 2:** Crystal structure of high-entropy ceramics.

Structure	Crystallography	Appearance	Ref.
Rock-salt	Cubic (Fm-3m)	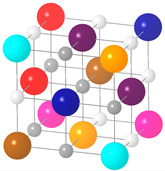	[112]
Fluorite	A_2_B_2_O_7_ cubic (Fm-3m)	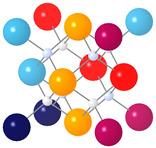	[113]
Perovskite	ABO_3_ Orthorhombic (Pbnm)	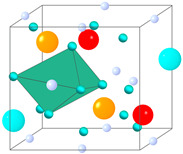	[114]
Perovskite	ABO_3_ Cubic (Pm3m)	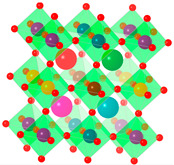	[115]
Perovskite	ABO_3_ Hexagonal (P63/mmc)	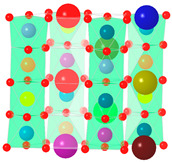	
Spinel	AB_2_O_4_ Cubic (Fd-3m)	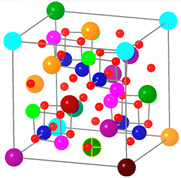	
Pyrochlore	A_2_B_2_O_7_ Cubic (Fd-3m)	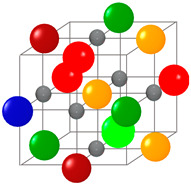	[116]
AlB_2_	Hexagonal (P6/mmm)	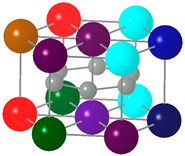	[111]

### 5.2. High-Entropy Effects in HE-UHTCs

According to the Gibbs free energy relationship, the change in Gibbs free energy of a thermodynamically stable single-phase solid solution at high-temperature can be described as:

(19) ΔG=ΔH−TΔS

where ΔG is the change in Gibbs free energy, ΔH is the enthalpy change, T is the absolute temperature, and ΔS is the entropy change. Stability in high-entropy ceramics does not mean that multicomponent ceramics remain thermodynamically stable at all temperatures. Rather, it reflects the competition between mixing enthalpy and configurational entropy. At high temperatures, the TΔS term increasingly lowers the Gibbs free energy and favors single-phase solid-solution formation. During cooling, however, the entropy contribution decreases, and local chemical ordering, short-range order, phase separation, or kinetic freezing may become dominant, potentially leading to local phase separation or entropy collapse. Therefore, so-called entropy stabilization in HE-UHTCs should be verified using in situ high-temperature X-ray diffraction (XRD), thermal-cycling phase analysis, CALculation of PHAse Diagrams (CALPHAD)/DFT free-energy calculations, and post-quenching phase-retention analysis, rather than inferred solely from the number of constituent elements [53].

As temperature increases, the contribution of entropy to Gibbs free energy becomes more significant, suppressing phase separation that would otherwise be promoted by a positive mixing enthalpy. Consequently, despite the extremely high degree of chemical disorder, simple crystal structures such as rock-salt carbides and hexagonal diborides remain the dominant structural motifs in HE-UHTCs. From a crystallographic perspective, significant atomic size mismatch and electronegativity differences among constituent elements make the stability of simple lattices in HE-UHTCs particularly remarkable. Severe lattice distortion emerges, generating heterogeneous local strain fields and distorted bonding environments. These distortions influence defect energetics, diffusion kinetics, and mechanical responses, thereby forming the structural basis for the unconventional properties observed in HE-UHTCs.

When four or more metallic elements with equimolar or near-equimolar compositions are incorporated into a system, the configurational entropy increases significantly, thermodynamically favoring the formation of simple solid-solution structures rather than multiphase coexistence. The elevated configurational entropy effectively reduces the Gibbs free energy of the system at high temperatures, thereby enhancing the thermodynamic stability of the solid-solution phase. This effect helps suppress the formation of undesirable brittle phases or low-melting-point phases, enabling the material to maintain a stable crystal structure under ultra-high-temperature conditions.

However, the applicability of the term “high-entropy” has been widely debated because the precise role of entropy in stabilizing these systems remains unclear [117]. There is no universally accepted standard defining whether a multicomponent system qualifies as high-entropy. Most high-entropy alloys are typically classified based on composition or configurational entropy. For a random solid solution, the configurational entropy per mole can be expressed as:

(20) $$\Delta S_{conf}=-R\sum_{i=1}^{n}\;X_{i}lnX_{i}$$

where $X_{i}$ is the molar fraction of component i and R is the gas constant. Based on combined definitions involving composition and entropy, a widely accepted classification has emerged: low entropy when S ≤ R, medium entropy when R ≤ S ≤ 1.5R, and high-entropy when S ≥ 1.5R [118].

The superior properties of HE-UHTCs do not originate from entropy maximization alone but rather from entropy-induced lattice distortion, sluggish diffusion, and multicomponent synergistic effects. Therefore, in the context of HE-UHTCs, the term “high-entropy” should be regarded primarily as a design strategy rather than a strict thermodynamic definition.

HE-UHTCs exhibit excellent mechanical and physical properties, high-temperature thermal stability, and superior oxidation resistance. Numerous studies on high-entropy borides, carbides, and nitrides have demonstrated superior hardness, wear resistance, and oxidation resistance compared with their single-component counterparts. In addition, increasing configurational entropy generally leads to reduced thermal conductivity in ceramic systems [119]. Furthermore, machine learning approaches combined with descriptor-based methods have been employed to predict entropy-forming ability and explore potential synthesis routes for high-entropy ceramics [120].

### 5.3. Lattice Distortion Effects in HE-UHTCs

In HE-UHTCs, random occupation of the same cation sublattice by multiple transition-metal elements introduces mass fluctuation, atomic-radius mismatch, local strain fields, and bond-strength variation. These local disorder effects cause mass fluctuation scattering, strain-field scattering, and force-constant-disorder scattering during phonon propagation, thereby shortening the phonon mean free path and reducing lattice thermal conductivity. For high-entropy carbides, nitrides, and diborides, lattice distortion not only modifies phonon dispersion and group velocity but may also further enhance phonon scattering through local short-range order, vacancies, and grain-boundary defects. Therefore, the effect of high-entropy design on thermal conductivity should primarily be understood as regulation of lattice thermal conductivity, rather than being simply equated with a decrease in total thermal conductivity [11,53].

Differences in atomic size, electronegativity, and chemical bonding characteristics among constituent elements can cause ions to deviate from their ideal sublattice positions, leading to lattice distortion. Unlike conventional high-entropy alloys, the increase in entropy in HE-UHTCs primarily arises from increased atomic disorder. Therefore, atomic size mismatch and mixing enthalpy are two primary criteria used to rationalize the formation of high-entropy solid solutions in multicomponent ceramic systems [111]. The average atomic size difference (δ) in multicomponent systems can be expressed as [121]:

(21) $$\delta =\sqrt{\overset{i=N}{\underset{i=1}{\sum }}X_{i}\left(1-\frac{r_{i}}{\sum_{i=1}^{i=N}X_{i}r_{i}}\right)}$$

where $X_{i}$ and $r_{i}$ represent the molar fraction and atomic radius of the i-th component, respectively. In HE-UHTCs, the atomic radii of constituent elements are generally similar, and the atomic size mismatch parameter typically satisfies δ ≤ 6.6% [122]. Similar to HEAs, the calculation of δ in HE-UHTCs mainly considers the contribution of metallic atoms while neglecting the contributions of B, C, and N atoms in borides, carbides, and nitrides. As a result, the actual lattice distortion in HE-UHTCs may be underestimated. In particular, differences in metallic atomic radii alone cannot fully describe the lattice distortion in boride systems.

Therefore, unlike HEAs, a more accurate representation of atomic size mismatch in multicomponent high-entropy ceramic systems can be obtained using the lattice constants of individual UHTC components:

(22) $$\delta_{a}=\sqrt{\overset{i=N}{\underset{i=1}{\sum }}X_{i}\left(1-\frac{a_{i}}{\sum_{i=1}^{i=N}X_{i}a_{i}}\right)}$$

(23) $$\delta_{c}=\sqrt{\overset{i=N}{\underset{i=1}{\sum }}X_{i}\left(1-\frac{c_{i}}{\sum_{i=1}^{i=N}X_{i}c_{i}}\right)}$$

where $a_{i}$ and $c_{i}$ are the measured lattice parameters of the i-th UHTC component. Another important parameter governing the formation of solid-solution phases is the mixing enthalpy ΔH_mix [123]:

(24) $$\Delta H_{mix}=\sum_{i=1;i\neq j}^{n}\;4\Delta H_{AB}^{mix}C_{i}C_{j}$$

**Figure 6:**
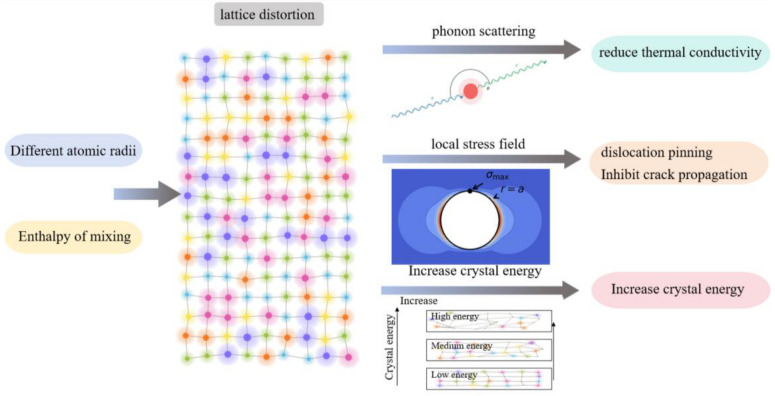
Schematic illustration of the lattice distortion effect in HE-UHTCs.

Lower formation enthalpy in HE-UHTCs indicates more favorable energetic conditions for the formation of single-phase high-entropy solid solutions [124]. Severe lattice distortion causes strong phonon scattering, which reduces the thermal conductivity of high-entropy ceramics. For example, the thermal conductivity of (Ce_0.2_Zr_0.2_Hf_0.2_Sn_0.2_Ti_0.2_)O_2_ is only about half that of 7 wt% yttria-stabilized zirconia. Recently, porous (Zr, Hf, Ti, Nb, Ta)B_2_ has been designed as a novel ultra-high-temperature thermal insulation material [125]. In ultra-high temperature thermal systems, thermal insulation materials are as important as heat-dissipating materials in preventing thermal damage. By optimizing processing conditions and parameters, highly porous structures suitable for thermal insulation can be tailored for HE-UHTCs [117]. Figure 6 shows the effect of lattice distortion on performance.

Severe lattice distortion in HE-UHTCs also generates local stress fields that hinder dislocation motion and crack propagation, leading to dislocation pinning. This mechanism contributes to increased hardness and strength and, in some cases, moderate improvements in fracture toughness. However, comprehensive data on bulk mechanical properties such as fracture strength and fracture toughness remain limited [117]. Additionally, lattice distortion increases the crystal energy, which suppresses grain coarsening by reducing the driving force for grain growth associated with surface energy reduction [126].

However, the influence of lattice distortion on toughness is not straightforward. Although crack deflection and energy dissipation may be promoted at the microscopic scale, chemical disorder may alter the dominant slip systems and thus influence ductility and hardness [127]. Excessive chemical disorder can also suppress dislocation mobility, limiting plastic accommodation. Therefore, entropy-induced strengthening does not necessarily translate into substantial improvements in toughness. The hardness of HE-UHTCs is generally higher than that of single-component UHTCs, and conventional rule-of-mixtures models are not applicable for predicting hardness in such multicomponent systems. Machine learning approaches have also been applied to predict lattice mismatch in high-entropy ceramics and to tune their elastic and mechanical properties [128,129].

### 5.4. Sluggish Diffusion Effects in HE-UHTCs

**Figure 7:**
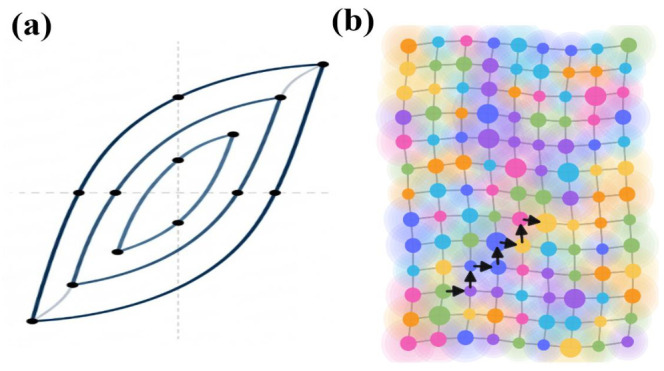
Schematic diagram of sluggish diffusion. (**a**) Each element cooperates with each other to slow down the diffusion rate. (**b**) Schematic diagram of diffusion lattice.

One of the most prominent consequences of high-entropy design is the so-called sluggish diffusion effect. In HE-UHTCs, multiple principal elements randomly occupy lattice sites in near-equimolar proportions, creating highly complex local chemical environments. Differences in atomic size, bonding characteristics, and electronegativity lead to pronounced lattice distortion and fluctuations in potential energy landscapes. As a result, atomic diffusion pathways become nonuniform, and migration energy barriers increase, hindering both cation and anion diffusion. Figure 7 shows the atomic delayed diffusion behavior.

Furthermore, chemical short-range order (CSRO) and multicomponent interactions significantly increase the activation energy required for atomic jumps and broaden the distribution of migration energy barriers, thereby reducing diffusion coefficients. Driven by the thermodynamics of mixing enthalpy, CSRO refers to the tendency of specific atoms to preferentially occupy nearest-neighbor positions and form locally ordered clusters at the nanoscale. These ordered regions act as asymmetric energy wells: the energy required for an atom to escape from an ordered region is much higher than that required to enter it, producing a pronounced atomic trapping effect and increasing the average residence time of atoms at specific lattice sites. In addition, atomic migration within CSRO regions often cannot proceed through single-atom jumps but requires cooperative multi-atom rearrangements, which substantially increases the effective activation energy. At the same time, CSRO clusters act as local energy minima, forcing diffusion paths to become more tortuous and increasing the effective diffusion distance. Macroscopically, this behavior appears as a non-Arrhenius decrease in diffusion coefficients and a significant reduction in the pre-exponential factor. This effect becomes particularly pronounced at high temperatures. Sluggish diffusion directly suppresses grain growth during sintering and thermal exposure [130,131], thereby enhancing microstructural stability and maintaining high strength at elevated temperatures.

From a functional perspective, reduced diffusion kinetics are crucial for maintaining mechanical integrity during prolonged high-temperature exposure. Compared with conventional UHTCs, HE-UHTCs exhibit improved resistance to grain coarsening and phase decomposition after long-term thermal exposure. Moreover, sluggish diffusion enhances high-temperature creep resistance and microstructural stability, enabling superior durability under prolonged service conditions.

In oxidation processes, the multicomponent cation sublattice creates a complex local chemical environment, broadening the distributions of atomic migration pathways and migration barriers. This may reduce long-range diffusion rates and delay phase separation or oxide scale growth. The thermal conductivity of high-entropy UHTCs can also be affected by oxygen impurity content. Introducing reducing components such as graphite can remove oxygen impurities, thereby increasing thermal conductivity and alleviating localized thermal gradients [132].

In irradiation environments, large numbers of point defects and dislocation loops are typically generated. Their aggregation and growth usually rely on diffusion-mediated migration. Sluggish diffusion reduces the effective mobility of these defects, suppressing the formation and evolution of defect clusters and thereby enhancing radiation tolerance in HE-UHTCs [133]. This diffusion-controlled behavior provides potential advantages for applications in extreme nuclear environments or high-energy particle irradiation conditions.

For HE-UHTCs, irradiation tolerance should be understood as a coupled defect-production, defect-migration and defect-annihilation problem. Energetic particles generate primary knock-on atoms (PKAs) and collision cascades, producing Frenkel pairs, vacancy, interstitial clusters, dislocation loops and stacking faults. Ramesh et al. showed that Frenkel-pair density can evolve through linear, exponential and plateau stages with increasing radiation dose, indicating that defect accumulation eventually approaches a saturation regime controlled by defect recombination and clustering. Their results also highlight the role of chemistry: higher Fe content increases vacancy formation energy and improves radiation resistance, whereas higher Cr content promotes diffusivity and defect accumulation due to a lower migration barrier. Aksoy et al. further demonstrated that amorphous interphase and grain-boundary complexions can markedly reduce residual radiation damage because excess free volume and interfacial disorder provide efficient defect recombination sites. Therefore, for HE-UHTCs, improved irradiation tolerance may arise from the combined effects of sluggish diffusion, lattice distortion, high defect-sink density, amorphous, nanostructured interphases and controlled grain-boundary chemistry [134,135].

However, the sluggish diffusion effect also presents challenges during material processing. Sintering densification relies on mass transport and pore elimination, and diffusion limitations significantly reduce the rate of densification, requiring longer sintering times and higher temperatures. Therefore, the fabrication of HE-UHTCs often relies on external-field-assisted techniques such as spark plasma sintering and hot pressing, or carefully designed sintering additive systems to overcome these processing limitations.

### 5.5. Cocktail Effect in HE-UHTCs

**Figure 8:**
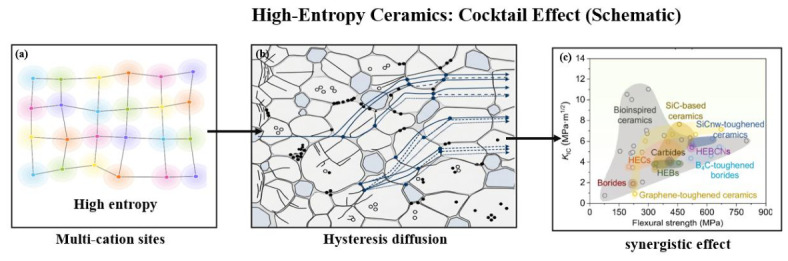
Schematic diagram of cocktail effect of high-entropy ceramics: (**a**) lattice distortion induced by high entropy, (**b**) atomic displacement associated with diffusion, and (**c**) fracture toughness KIC and flexural strength of HEC/Cr7C3 all-ceramics compared to those of other reported structural ceramics. The colored shadings represent different categories of ceramic materials as labeled, and the circles indicate the specific values of these materials. Reproduced from [139], under the terms of the Creative Commons Attribution-NonCommercial-NoDerivatives 4.0 International License (CC BY-NC-ND 4.0).

The cocktail effect should be understood as a nonlinear property response caused by multielement coupling rather than a simple superposition of multiple elements. In HE-UHTCs, metal–nonmetal bond strength, electronic structure, lattice distortion, defect chemistry, phase stability, and microstructural evolution are coupled, which may cause hardness, elastic modulus, fracture toughness, oxidation resistance, thermal conductivity, or melting point to deviate from simple rule-of-mixtures predictions [53]. Figure 8 shows the changes in material properties caused by the cocktail effect.

Chemical complexity promotes the formation of multicomponent oxide scales with higher thermodynamic stability and lower volatility. These complex oxides generally exhibit higher viscosity, lower oxygen diffusivity, and stronger adhesion compared with binary oxides. Solid-solution strengthening effects arising from the mutual dissolution of different metallic elements have been associated with improved hardness and fracture toughness [136]. The cocktail effect arising from multicomponent coupling can simultaneously enhance strength and fracture toughness in selected HE-UHTCs.

For example, the addition of Mo has been reported to promote grain refinement and achieve fine-grain strengthening, whereas the addition of K can facilitate densification during ceramic sintering. The synergistic effect of these mechanisms improves the fracture toughness of HE-UHTCs [137,138].

In high-entropy carbides and diborides, selective oxidation may occur during the early stages of oxidation. Elements that form thermodynamically stable oxides, such as HfO_2_ and ZrO_2_, preferentially oxidize to form refractory barrier layers. Meanwhile, other elements can generate glassy or semi-glassy phases that seal microcracks and pores. This cooperative oxidation behavior allows HE-UHTCs to maintain protective surface layers over broader temperature and flow conditions. High-entropy diborides generally exhibit better oxidation resistance than the average performance of their single-metal diboride counterparts [111]. (Al_0.34_Cr_0.22_Nb_0.11_Si_0.11_Ti_0.22_)_50_N_50_ showed a weight gain of only 0.015 mg·cm^−2^ after oxidation at 1573 K for 50 h [140]. Using a pulsed current processing method, B_4_(HfMo_2_TaTi)C + SiC exhibited less than 1% weight loss after oxidation at 1173 K for 50 h [124]. Furthermore, the addition of SiC can significantly improve oxidation resistance by forming a protective SiO_2_ layer on the sample surface.

Wang et al. [141] investigated the oxidation resistance of (Hf_0.2_Zr_0.2_Ta_0.2_Nb_0.2_Ti_0.2_)C mixed with different amounts of SiC (10, 20, and 30 vol%) in the temperature range of 1573–1773 K. Due to the formation of protective complex oxides such as HfZrSiO_4_ and HfZrTiO_4_, dense oxide layers were formed without reacting with the underlying substrate. These results highlight the importance of understanding the formation and evolution of complex oxides during oxidation of multicomponent HE-UHTCs. Although the addition of SiC does not fundamentally change the oxidation mechanism, it delays the outward diffusion of other elements. The sample containing 20 vol% SiC exhibited the best oxidation resistance.

Gild et al. [111] synthesized high-entropy diboride ceramics via ball milling and spark plasma sintering and reported hardness values higher than those of conventional diborides. However, ball milling may introduce pores and oxides into the structure, which can negatively affect hardness. They synthesized several compositions, including (Hf_0.2_Zr_0.2_Ta_0.2_Nb_0.2_Ti_0.2_)B_2_, (Hf_0.2_Zr_0.2_Ta_0.2_Mo_0.2_Ti_0.2_)B_2_, (Hf_0.2_Zr_0.2_Mo_0.2_Nb_0.2_Ti_0.2_)B_2_, (Hf_0.2_Mo_0.2_Ta_0.2_Nb_0.2_Ti_0.2_)B_2_, (Mo_0.2_Zr_0.2_Ta_0.2_Nb_0.2_Ti_0.2_)B_2_, and (Hf_0.2_Zr_0.2_Ta_0.2_Cr_0.2_Ti_0.2_)B_2_, and investigated their oxidation behavior. The results showed that all of these high-entropy borides exhibited better oxidation resistance than their corresponding binary borides (except HfB_2_). In addition, (Hf_0.2_Mo_0.2_Zr_0.2_Nb_0.2_Ti_0.2_)B_2_ prepared by reactive spark plasma sintering exhibited excellent oxidation resistance at temperatures up to 1473 K for 6 h [142]. Its oxidation behavior was similar to that of oxidation-resistant HfB_2_ but superior to that of most other binary diborides.

To date, oxidation studies of HE-UHTCs reported in the literature have largely been limited to furnace oxidation experiments. However, to make these materials suitable for real ultra-high temperature environments, dynamic oxidation testing is also required.

Finally, it should be noted that oxidation resistance still depends strongly on composition. Excessive incorporation of elements that form volatile oxides or low-melting-point phases can weaken the protective oxide layer. Therefore, high-entropy design must be guided by oxide thermodynamics and diffusion kinetics rather than relying solely on entropy maximization.

### 5.6. Comparative Properties of HE-UHTCs

It should be noted that processing routes, relative density, grain size, second-phase content, applied load, oxidation atmosphere, and testing temperature differ among the cited studies. Therefore, the table should not be interpreted as an absolute ranking, but rather as a direct comparison of property ranges and design trends among different material systems. Overall, HE-UHTCs generally exhibit higher hardness or lower thermal conductivity and, in some systems, improved oxidation stability. However, their fracture toughness and ablation resistance remain strongly dependent on density, second phases, oxide scale composition, and testing conditions. Table 3 presents commonly used high entropy ceramics for ultra-high temperature applications.

**Table 3:** Properties of selected high-entropy ceramics.

High-Entropy Carbide	Preparation Condition	Crystal Structure	Vickers Hardness (GPa)	Elastic Modulus (GPa)	Fracture Toughness (MPa·m^1/2^)	Reference
Hf_0.2_Ta_0.2_Ti_0.2_Nb_0.2_Zr_0.2_C	—	FCC	25.7 ± 3.5	473 ± 37	—	[143]
Hf_0.2_Ta_0.2_Ti_0.2_Nb_0.2_Mo_0.2_C	—	FCC	23.8 ± 2.7	544 ± 48	—	[143]
(Hf_0.2_Zr_0.2_Ta_0.2_Nb_0.2_Ti_0.2_)C	SPS	Rock-salt	17.07 ± 0.54 (9.8 N)	—	5.9 ± 0.7	[144]
(Hf_0.2_Zr_0.2_Ta_0.2_Nb_0.2_Ti_0.2_)C	PLS	—	17.7 ± 0.5 (9.8 N)	—	4.3 ± 0.2	[145]
(TiZrHfNbTaMo)C	PLS	—	23.2 (9.8 N)	—	3.7 ± 0.2	[146]
(Hf,Zr,Ti,Ta,Nb)C	CTR	Rock-salt	24.8 ± 0.8 (4.9 N)	452 ± 6	—	[105]
(Hf_0.2_Ta_0.2_Zr_0.2_Nb_0.2_Ti_0.2_)C	HP	—	24	—	2.306	[147]
(Hf–Ta–Zr–Nb)C	SPS	Rock-salt	36.1 ± 1.6	598 ± 15	—	[148]
(NbTaZrTiHfVWMo)C	—	—	38.71	—	4.67	[149]
(Ti_0.2_Zr_0.2_Hf_0.2_Nb_0.2_Ta_0.2_)C + 20 vol.% SiC	SPS	FCC	25 ± 0.8 (9.8 N)	—	5.24 ± 0.41	[150]
(Ti_0.2_Zr_0.2_Hf_0.2_Nb_0.2_Ta_0.2_)C	SPS	—	21.9 ± 0.4 (9.8 N)	—	4.51 ± 0.61	[150]
(TiZrNbTaMo)C	HP	—	25.3 (9.8 N)	—	3.28	[137]
(Ti_0.2_Zr_0.2_Hf_0.2_Ta_0.2_Nb_0.2_)C	SPS	—	20.39 (9.8 N)	—	4.5 ± 0.6	[151]
(TiZrHfVNbTa)C	—	—	24.9 (9.8 N)	—	—	[152]
(VNbTaMoW)C_5_	SPS	—	19.6	—	5.4	[153]
(VNbTaMoW)C	SPS	Rock-salt	23.8	—	3.34	[154]
(Zr_0.25_Nb_0.25_Ti_0.25_V_0.25_)C	—	Rock-salt	30.3 ± 0.7	460.4 ± 19.2	4.7 ± 0.5	[136]
(Zr_0.25_Hf_0.25_Ta_0.25_Nb_0.25_)C	SPS	—	—	—	4.73	[155]
High-entropy nitrides						
(Al_29.1_Cr_30.8_Nb_11.2_Si_7.7_Ti_21.2_)N_x_	RMS	FCC (NaCl-type)	36.7	—	—	[156]
(Al_23.1_Cr_30.8_Nb_7.7_Si_7.7_Ti_30.7_)N_50_	RMS	FCC (NaCl-type)	36.1	—	—	[156]
(AlCrNbSiTiV)N	RMS	FCC (NaCl-type)	41	30	—	[157]
(AlCrTiZrHf)N	RMS	FCC	33.1	—	—	[158]
(Al_0.34_Cr_0.22_Nb_0.11_Si_0.11_Ti_0.22_)_50_N_50_	RMS	FCC (NaCl-type)	36 (5 mN)	—	—	[159]
(Al,Ta,Ti,V,Zr)N	RMS	—	~20	433	2.4	[160]
(AlCrNbSiTiV)N	RMS	FCC (NaCl-type)	>40	—	—	[126]
(AlCrTiZrHf)N	RMS	FCC	33.1	—	—	[158]
(CrHfTaTiZr)_1_N_x_	—	FCC (NaCl-type)	26.5	465.5	—	[161]
(CrHfNbTiZr)_1_N_x_	—	FCC	27.3	488.4	—	[161]
(CrHfNbTaTi)_1_N_x_	—	FCC	26.2	488.9	—	[161]
(CrNbTaTiV)1N_x_	—	FCC	24.4	476.7	—	[161]
(Hf,Ta,Ti,V,Zr)N	RMS	FCC (NaCl-type)	32.5 ± 0.8	—	—	[162]
Hf–Nb–Ti–V–Zr–N	RMS	FCC	18.8	418	—	[163]
(Hf_0.2_Nb_0.2_Ta_0.2_Ti_0.2_Zr_0.2_)N	SPS	FCC	Up to 33 (4.9 N)	—	Up to 5.2	[164]
(MoSiTiVZr)N_x_	RMS	—	45.6	—	—	[165]
(HfNbTaTiZr)1N_x_	—	FCC (NaCl-type)	27.8	502.6	—	[161]
(TiZrNbHfTa)N/WN	VAC	FCC	34	325	—	[166]
(TiVCrZrNbMoHfTaWAlSi)N	RMS	FCC + Hexagonal	34.8	—	—	[167]
High-entropy boride						
(Hf_0.2_Zr_0.2_Ta_0.2_Nb_0.2_Ti_0.2_)B_2_	SPS	Hexagonal	17.5 ± 1.2 (1.96 N)	—	—	[111]
(Hf_0.2_Zr_0.2_Ta_0.2_Mo_0.2_Ti_0.2_)B_2_	SPS	Hexagonal	19.1 ± 1.8 (1.96 N)	—	—	[111]
(Hf_0.2_Zr_0.2_Mo_0.2_Nb_0.2_Ti_0.2_)B_2_	SPS	Hexagonal	21.9 ± 1.7 (1.96 N)	—	—	[111]
(Hf_0.2_Mo_0.2_Ta_0.2_Nb_0.2_Ti_0.2_)B_2_	SPS	Hexagonal	22.5 ± 1.7 (1.96 N)	—	—	[111]
(Hf_0.2_Zr_0.2_Ta_0.2_Cr_0.2_Ti_0.2_)B_2_	SPS	Hexagonal	21.0 ± 2.8 (1.96 N)	—	—	[111]
(Hf_0.2_Mo_0.2_Ta_0.2_Nb_0.2_Ti_0.2_)B_2_	SPS	Hexagonal	22.5 ± 1.7 (1.96 N)	—	—	[168]
(Hf_0.2_Zr_0.2_Ti_0.2_Ta_0.2_Mo_0.2_)B_2_	BCR	Hexagonal	24.9 ± 1.0 (1.96 N)	—	—	[169]
(Hf_0.2_Zr_0.2_Ti_0.2_Ta_0.2_Nb_0.2_)B_2_	BCR	Hexagonal	20.5 ± 1.0 (1.96 N)	—	—	[169]
(Hf_0.2_Zr_0.2_Ti_0.2_Ta_0.2_Cr_0.2_)B_2_	BCR	Hexagonal	24.9 ± 1.0 (1.96 N)	—	—	[169]
(Hf_0.2_Zr_0.2_Ta_0.2_Nb_0.2_Ti_0.2_)B_2_	SPS	Hexagonal	21.7 ± 1.1 (1.96 N)	—	4.06 ± 0.35	[170]
(Hf_0.2_Zr_0.2_Mo_0.2_Nb_0.2_Ti_0.2_)B_2_	SPS	Hexagonal	26.3 ± 1.8 (1.96 N)	—	3.64 ± 0.36	[170]
(Hf_0.2_Mo_0.2_Ta_0.2_Nb_0.2_Ti_0.2_)B_2_	SPS	Hexagonal	27.0 ± 0.4 (1.96 N)	—	4.47 ± 0.40	[170]
(Hf_0.2_Zr_0.2_Ta_0.2_Cr_0.2_Ti_0.2_)B_2_	SPS	Hexagonal	28.3 ± 1.6 (1.96 N)	—	—	[171]
(Hf_0.2_Mo_0.2_Zr_0.2_Nb_0.2_Ti_0.2_)B_2_	SPS	Hexagonal	26.3 ± 0.7 (1.96 N)	—	—	[171]
(Hf_0.2_Zr_0.2_Ta_0.2_Cr_0.2_Ti_0.2_)B_2_	SPS	Hexagonal	29.3 (1.96 N)	—	3.56	[172]
(Hf_0.2_Zr_0.2_Ti_0.2_Ta_0.2_Cr_0.2_)B_2_	AC	—	22.6 (1.96 N)	—	—	[173]
(Mo_0.2_Zr_0.2_Ta_0.2_Nb_0.2_Ti_0.2_)B_2_	SPS	Hexagonal	23.7 ± 1.7 (1.96 N)	—	—	[111]
(Ti_0.2_Hf_0.2_Zr_0.2_Nb_0.2_Ta_0.2_)B_2_	BCR	Hexagonal	25.6 ± 0.8	500	2.83 ± 0.15	[174]
(Ti_0.2_Zr_0.2_Hf_0.2_Mo_0.2_W_0.2_)B_2_	BCR	Hexagonal	26.0 ± 1.5 (1.96 N)	—	—	[175]
(Ti_0.2_Ta_0.2_Cr_0.2_Mo_0.2_W_0.2_)B_2_	BCR	Hexagonal	23.7 ± 1.3 (1.96 N)	—	—	[175]
(Ti_0.2_Zr_0.2_Hf_0.2_Nb_0.2_Ta_0.2_)B_2_	BCR	Hexagonal	20.9 ± 1.3 (1.96 N)	505	3	[175]
(Ti_0.2_Zr_0.2_Hf_0.2_Nb_0.2_Ta_0.2_)B_2_	HP	—	23.7 ± 0.7 (1.96 N)	—	3.81 ± 0.40	[176]
(Ti_0.2_Zr_0.2_Hf_0.2_Nb_0.2_Ta_0.2_)B_2_-20vol.% SiC	HP	—	24.8 ± 1.2 (1.96 N)	—	4.85 ± 0.33	[176]
(Zr_0.2_Hf_0.2_Nb_0.2_Ta_0.2_W_0.2_)B_2_	BCR	Hexagonal	26.7 ± 1.1 (1.96 N)	—	—	[175]
(Zr_0.23_Ti_0.20_Hf_0.19_V_0.14_Ta_0.24_)B_2_	NRMS	Hexagonal	47.2 ± 1.8	540.1 ± 17.1	—	[118]

Notes: The “Preparation condition” column lists the primary densification or deposition method used to fabricate the specimen. Abbreviations: SPS: Spark Plasma Sintering. HP: Hot Pressing. PLS: Pressureless Sintering. CTR: Carbothermal Reduction (followed by densification). BCR: Borocarbothermal Reduction (followed by densification). RMS: Reactive Magnetron Sputtering. NRMS: Non-reactive Magnetron Sputtering. VAC: Vacuum Arc deposition. AC: Arc-melting. “—”: not reported or not specified. Vickers hardness values are reported at the indentation load indicated in parentheses. Direct numerical comparison between entries tested at different loads should be made with caution, as hardness values typically decrease with increasing load due to the indentation size effect. Properties listed correspond to dense, bulk specimens or coatings as fabricated. values may vary depending on starting powder purity, relative density, grain size, and second-phase content.

As shown in Table 4, the advantages of HE-UHTCs do not arise from simultaneous improvement in all properties, but rather from a redistribution of property profiles. High-entropy carbides and nitrides often exhibit higher hardness than conventional carbides and nitrides. For example, (Zr_0.25_Nb_0.25_Ti_0.25_V_0.25_)C has a hardness of 30.3 GPa, higher than most conventional carbides. Some high-entropy nitrides can reach hardness values of 36–45.6 GPa. The fracture toughness of high-entropy carbides and borides is commonly around 4–6 MPa·m^1/2^, which is higher than that of many monolithic carbides and ZrB_2_/HfB_2_, although not necessarily higher than TiB_2_ or continuous-fiber-reinforced composites. High-entropy design typically enhances mass fluctuation, atomic-size-mismatch, and force-constant-disorder scattering, thereby reducing lattice thermal conductivity. For instance, (Zr_0.25_Nb_0.25_Ti_0.25_V_0.25_)C has a room-temperature thermal conductivity of approximately 15.3 W·m^−1^·K^−1^, lower than that of most conventional diborides and some carbides. The oxidation-resistance advantage of HE-UHTCs mainly originates from multicomponent oxide scales, sluggish diffusion, and cooperative protection by complex oxides, rather than solely from increased configurational entropy. For example, high-entropy borides and SiC-containing high-entropy carbides can form protective scales containing HfO_2_, ZrO_2_, TiO_2_, SiO_2_, or complex silicates, thereby improving oxide scale density and volatility resistance. A current limitation is that many HE-UHTCs still lack thermal conductivity, oxidation-onset temperature, and long-term ablation data measured under identical testing conditions. Therefore, standardized datasets should be established to enable rigorous quantitative comparison between conventional UHTCs and HE-UHTCs.

**Table 4:** Key property comparison between representative conventional UHTCs and HE-UHTCs.

Category	Representative Composition	Crystal Structure	Hardness (GPa)	Fracture Toughness (MPa·m^1/2^)	Oxidation Temperature or Behavior	Thermal Conductivity (W·m^−1^·K^−1^)	Refs.
Conventional carbide	HfC	FCC	24.2	—	Forms HfO_2_; refractory oxide may provide short-term protection.	22.2	[23,62,72]
Conventional carbide	ZrC	FCC	25	—	Forms ZrO_2_; C escapes as CO/CO_2_ and may introduce pores.	20.61	[23,54,62,72]
Conventional carbide	TiC	FCC	23.6	4	Forms TiO_2_ at high temperature; limited oxidation protection.	17–21	[23,62]
Conventional nitride	TiN	FCC	18.6	—	N may escape as N_2_ during oxidation, reducing oxide scale integrity.	29.1	[23,62]
Conventional nitride	ZrN	FCC	15	—	Forms ZrO_2_; nitrogen release may generate porosity.	20.9	[23,62,63]
Conventional boride	TiB_2_	Hexagonal	25–35	5–7	B_2_O_3_ can seal pores at intermediate temperature but volatilizes at high temperature.	60–120	[64,65]
Conventional boride	ZrB_2_	Hexagonal	13.7	3.1	Near-parabolic oxidation at 945–1256 °C; B_2_O_3_ volatilization dominates above 1400 °C.	60–130	[66,67,74,76,177]
Conventional boride	HfB_2_	Hexagonal	18	3.1	Oxidation accelerates near 1627 °C.	—	[68,75]
HE carbide	(Zr_0.25_Nb_0.25_Ti_0.25_V_0.25_)C	Rock-salt	30.3 ± 0.7	4.7 ± 0.5	Not systematically reported.	15.3 ± 0.3	[136]
HE carbide	(Hf_0.2_Zr_0.2_Ta_0.2_Nb_0.2_Ti_0.2_)C	Rock-salt	17.07 ± 0.54	5.9 ± 0.7	Not systematically reported.	—	[144]
HE carbide + SiC	(Ti_0.2_Zr_0.2_Hf_0.2_Nb_0.2_Ta_0.2_)C + 20% SiC	FCC	25 ± 0.8	5.24 ± 0.41	Best oxidation resistance among 10–30 vol.% SiC samples at 1573–1773 K.	—	[141,150]
HE nitride	(Al_0.34_Cr_0.22_Nb_0.11_Si_0.11_Ti_0.22_)_50_N_50_	NaCl-type	36	—	Mass gain of only 0.015 mg·cm^−2^ after oxidation at 1573 K for 50 h.	—	[140,159]
HE boride	(Hf_0.2_Zr_0.2_Ta_0.2_Nb_0.2_Ti_0.2_)B_2_	Hexagonal	21.7 ± 1.1	4.06 ± 0.35	HE borides generally show better oxidation resistance than most corresponding binary diborides.	—	[111,170]
HE boride	(Hf_0.2_Mo_0.2_Ta_0.2_Nb_0.2_Ti_0.2_)B_2_	Hexagonal	27.0 ± 0.4	4.47 ± 0.40	Not systematically reported.	—	[170]
HE boride + SiC	(Ti_0.2_Zr_0.2_Hf_0.2_Nb_0.2_Ta_0.2_)B_2_20 vol.% SiC	—	24.8 ± 1.2	4.85 ± 0.33	SiC can support formation of silicate glass protection.	—	[176]
HE boride	(Hf_0.2_Mo_0.2_Zr_0.2_Nb_0.2_Ti_0.2_)B_2_	Hexagonal	26.3 ± 0.7	—	Good oxidation resistance after exposure at 1473 K for 6 h.	—	[142,171]
HE boride	(Zr_0.23_Ti_0.20_Hf_0.19_V_0.14_Ta_0.24_)B_2_	Hexagonal	47.2 ± 1.8	—	Not systematically reported.	—	[118]

Notes: This table is intended as a qualitative comparison of representative property ranges and design trends between conventional UHTCs and HE-UHTCs. It should not be interpreted as an absolute performance ranking. “—” indicates data not reported or not available under comparable testing conditions. Hardness values correspond to Vickers indentation measurements. Indentation loads differ among cited studies and are not uniformly specified. Fracture toughness values were obtained by different methods across studies, and cross-method comparison may introduce systematic discrepancy. Thermal conductivity values are reported at or near room temperature unless otherwise stated. Oxidation behavior descriptions are summarized from individual studies conducted under varying atmospheres, temperatures, and exposure durations. They do not represent standardized or directly comparable test results. Processing routes, relative density, grain size, and second-phase content differ among cited studies and critically influence all listed properties.

Although high-entropy design has been widely used to improve the phase stability, thermal conductivity, and mechanical properties of UHTCs, clear comparability limitations remain among existing studies. Differences in processing routes, sintered density, grain size, nonstoichiometry, second-phase content, and testing temperature make direct numerical comparison of properties potentially misleading. In addition, the sluggish diffusion and cocktail effects are still inferred indirectly in many studies, with limited direct evidence such as diffusion coefficients, activation energies, deviations from rule-of-mixtures behavior, or in situ high-temperature characterization. Future HE-UHTC research should therefore shift from simply reporting new compositions and high performance values toward verifiable mechanisms, comparable datasets, and predictable design [53].

The final properties of HE-UHTCs depend not only on compositional design but also strongly on the sintering route, residual porosity, grain-boundary chemistry, and non-equilibrium phase formation during rapid densification. HP generally relies on high temperature, long dwell time, and sustained external pressure to promote mass diffusion and pore closure, which favors high relative density but can also cause grain coarsening, grain-boundary segregation, and second-phase growth. In contrast, SPS features high heating rates, short dwell times, and field-assisted densification, allowing grain growth to be suppressed and high density to be achieved within a short time; consequently, it is widely used to fabricate HE-UHTCs. However, the rapid non-equilibrium nature of SPS may also lead to insufficient elemental interdiffusion, local chemical heterogeneity, incomplete removal of residual oxides, and retention of metastable or intermediate reaction phases. Wyatt et al. pointed out that the sinterability of UHTCs is first controlled by the oxygen content, inorganic impurities, and particle size of the starting powders. Native oxide layers hinder densification and limit high-temperature thermomechanical properties, whereas smaller particle size and higher powder purity favor near-full densification at lower temperatures [178,179]. For example, the thermal conductivity of ZrB_2_ can increase from approximately 80 W·m^−1^·K^−1^ to approximately 150 W·m^−1^·K^−1^ depending on the powder synthesis route and purity, showing that processing conditions and impurity control can significantly alter final properties rather than merely phase composition [2,179]. In addition, the strong covalent M–B and M–C bonds in UHTCs lead to low grain-boundary and lattice diffusion rates, making densification difficult. Additives such as SiC, B_4_C, MoSi_2_, metals, and rare-earth oxides are therefore commonly used to promote liquid-phase sintering, increase diffusion rates, or inhibit grain growth [64,180,181]. However, these grain-boundary phases and additives have dual effects: they can promote densification, close pores, and improve oxide scale continuity, but soft glassy phases, residual oxides, silicides, or low-melting phases may soften, decompose, or segregate at high temperature, thereby reducing elastic modulus, yield strength, and fracture toughness [182,183]. Therefore, processing parameters such as SPS or HP temperature, pressure, heating rate, dwell time, powder particle size, and oxygen content determine relative density, open porosity, grain size, grain-boundary phases, and second-phase distribution, and are critical to the properties of UHTCs.

## 6. Artificial Intelligence-Accelerated Design of UHTCs

The design and optimization of UHTCs have traditionally been constrained by the vast compositional space, complex phase equilibria, and the extreme experimental conditions required for validation. In recent years, artificial intelligence (AI) and machine learning (ML), initially regarded as supplementary tools to conventional computational approaches, have gradually evolved into key technological pathways for overcoming the design bottlenecks of UHTCs. Their central advantage lies in the rapid establishment of data-driven mappings among composition, structure, processing, and properties.

### 6.1. Machine Learning for UHTC Design

Conventional experimental methods are mainly suitable for exploring low-dimensional compositional spaces, such as binary, ternary, or additive-containing systems [184,185], whereas ML is better suited for high-dimensional compositional searches, such as five-component, six-component, multicomponent, and multiple processing-parameter combinations in high-entropy ceramics. Machine learning models can dynamically adjust input variables and generate rapid and accurate predictions under diverse conditions, thereby substantially accelerating the UHTC design cycle [54]. Existing ML studies have mainly focused on HfB_2_, ZrB_2_, and additive-containing systems. Kaufmann et al. [186] were the first to introduce ML methods into the design of high-entropy ceramics. Using decision-tree models, they predicted the entropy forming ability (EFA) of HECs, and the results showed strong agreement with DFT calculations.

A typical ML workflow includes data collection, database construction, feature engineering, model training and selection, model application, and experimental validation. The success of ML studies depends fundamentally on the availability of suitable datasets. To date, ML techniques have already been successfully applied in UHTCs research for both property prediction and materials design [184]. By integrating physical models with data-driven approaches, researchers have predicted the thermal load-bearing capacity and ablation resistance of ultra-high-temperature ceramics [187]. ML has also been used to predict Young’s modulus, flexural strength, fracture toughness [188], and oxide scale thickness [189,190].

The descriptors employed in ML for materials design typically include elemental properties, such as atomic radius, electronegativity, and melting point; thermodynamic parameters, such as mixing enthalpy and configurational entropy; and structural features, such as crystal structure type and lattice distortion. These descriptors serve as inputs to supervised learning models in order to establish quantitative relationships between composition and target properties.

It should be noted that ML models for UHTCs and HE-UHTCs should use descriptors selected according to the target property. For hardness and mechanical properties, effective descriptors generally include VEC, shear modulus *G*, bulk modulus B, Pugh’s ratio G/B, Young’s modulus, bond strength, lattice-distortion parameters, and electronic deviations among constituent elements. For example, in high-throughput studies of Hf-Ta-C-N systems, Vickers hardness can be semi-quantitatively estimated using the Tian formula [191]:

(25) $$H_{V}=0.92{\left(G/B\right)}^{1.137}G^{0.708}$$

The formula can be used for semi-quantitative estimation, and carbonitrides exhibit relatively high shear stiffness near the corresponding optimized composition range such as VEC≈8.3–8.5, indicating a coupling among VEC, elastic modulus, shear resistance, and hardness [54,191]. For oxidation and ablation resistance, descriptors should extend beyond composition alone to include oxidation reaction enthalpy, formation energy, melting point, density, coating thickness, porosity, transition layers, testing temperature, oxygen gas flow rate, ablation distance, and exposure time. Hao et al. developed a random forest regression (RFR) model for the mass ablation rate of UHTC-coated C/C composites. The model achieved MAE = 0.55 and MSE = 0.71 and R^2^ = 0.87 on the test set. SHapley Additive exPlanations (SHAP) analysis showed that the most important features were the minimum melting point among coating constituent phases $Mp_{\min}$, the weighted average oxidation reaction enthalpy of non-oxide components $Hfo_{\mathrm{ave}}$, the weighted average formation energy of coating phases $Fe_{\mathrm{ave}}$, the distance between the specimen and the torch during ablation testing $T_{d}$, and the specimen surface temperature during ablation testing $T_{em}$. The mass ablation rate increased with increasing $Mp_{\min}$, $Hfo_{\mathrm{ave}}$, $Hf_{\mathrm{ave}}$ and $Fe_{\mathrm{ave}}$. whereas the mass ablation rate decreased with increasing the density of the highest-density constituent phase in the coating $\rho_{\max}$ and the molar-weighted average density of the coating constituent phases $\rho_{\mathrm{ave}}$. These results indicate that a high-density, low-porosity structure is more effective in reducing ablation loss than simply introducing high-melting-point phases [192]. Bianco et al. developed an ML model for oxidation damage in Hf/Zr/Ta carbides using composition, mean grain size, relative densification, holding time, and temperature as descriptors. The model predicted oxide-scale thickness with an MAE of ±65.45 μm. However, when the recession rate exceeded approximately 60 μm·min^−1^, the error increased to ±134.34 μm, indicating that data remain insufficient under rapid spallation, volume expansion, and extreme ablation conditions [184]. For phase stability, disordered enthalpy-entropy descriptor (DEED), $\Delta H_{\mathrm{hull}}$, formation-enthalpy distribution width, configurational entropy, and compensation temperature $\Theta ={\left(k_{B}DEED\right)}^{-1}$ are more suitable than VEC alone as descriptors for single-phase solid-solution synthesizability. Divilov et al. proposed the DEED, which simultaneously considers the entropy gain associated with forming disordered solid solutions and the enthalpy cost relative to stable phases on the convex hull. This descriptor is better suited for predicting the functional synthesizability of high-entropy carbides, carbonitrides, and borides and can be expressed as:

(26) $$\mathrm{DEED}=\frac{\sigma^{-1}{\left[H_{f}\right]}_{\Omega }}{{\langle \Delta H_{\mathrm{hull}}\rangle }_{\Omega }}$$

where Ω represents the thermodynamic density of states of the disordered system, $H_{f}$ represents the formation enthalpy of each configuration, and $\Delta H_{\mathrm{hull}}$ represents the energy distance of each configuration from the convex hull. The entropy-related term describes the entropy gain associated with forming disordered configurations $\sigma^{-1}{\left[H_{f}\right]}_{\Omega }$, whereas the enthalpy-related term describes the average enthalpy penalty relative to thermodynamically stable convex-hull phases ${\langle \Delta H_{\mathrm{hull}}\rangle }_{\Omega }$. Therefore, DEED is essentially an entropy gain/enthalpy cost balance descriptor and can more directly reflect the thermodynamic driving force for single-phase high-entropy ceramic formation than VEC alone [120]. Kim et al. constructed temperature and nitrogen-partial-pressure-dependent phase diagrams for the Hf-Ta-C-N quaternary system using ab initio thermodynamics. They found that solid solutions occupy a large phase space at relatively low temperatures, but as temperature increases, the stable compositional region shifts from the Ta/N-rich side toward the Hf/C-rich side, confirming high phase stability of (Hf_0.2_Ta_0.8_)C. Therefore, data-driven screening of HE-UHTCs for extreme service environments should include temperature, atmosphere, $\Delta H_{hull}$, stable regions in phase diagrams, and nonstoichiometry as phase-stability descriptors [191].

Current ML models for UHTCs are still limited by small datasets, inconsistent testing methods, and missing key variables. Although Han et al.’s mechanical-property model integrated data from 32 publications, the datasets for Young’s modulus, flexural strength, and fracture toughness contained only 122, 182, and 104 data points, respectively. The fracture-toughness model showed the lowest accuracy, mainly because of small data volume and inconsistent testing methods, including four-point bending, chevron-notch beam, Vickers indentation, and single-edged notch beam methods. Key variables such as residual stress are also often not reported [188]. Hao et al.’s ablation model for UHTC coatings retained only 123 complete samples after cleaning 159 experimental samples and required treatment of missing values, outliers, and dimensional normalization [192]. Bianco et al.’s carbide oxidation model contained only 76 data points, with fewer than 10 samples representing rapid volume expansion; therefore, the model error increased significantly when the recession rate exceeded approximately 60 μm·min^−1^ [184]. These results indicate that ML models in the UHTC field are currently more suitable as candidate prescreening tools than as high-precision substitutes for experimental validation. Future databases should standardize records of composition, sintering conditions, relative density, grain size, porosity, testing temperature, oxygen partial pressure, heat flux, exposure time, testing method, and uncertainty range, and should evaluate model generalization using external test sets or cross-literature validation whenever possible.

As a powerful data-driven methodology, ML has recently become one of the most influential modeling tools in materials science. Although ML methods have been successfully applied to multicomponent UHTCs for tasks such as phase prediction and mechanical-property prediction [193], only a limited number of studies have attempted to use ML for oxidation prediction in relatively simple ceramic systems [189]. A major challenge arises from the severe scarcity of data and the variable quality of existing datasets. To address this issue, researchers have proposed methods such as data augmentation, generative adversarial networks (GANs), and transfer learning. For example, data-augmented GANs (DAGANs) have been used to predict hardness, elastic modulus, and wear resistance in high-entropy nitride ceramics, with the generated samples improving the robustness of the models [194].

In addition, by integrating structural and thermodynamic parameters, ML can be used to predict the formation of single-phase solid solutions in multicomponent ultra-high-temperature ceramics [195]. Physics-based multiscale methods can also generate high-quality input datasets, which are essential for the successful application of ML techniques in materials research [185].

To bridge the gap between the computational cost of first-principles calculations and the predictive limitations of traditional ML models, a new class of methods has emerged in which ML is used to construct interatomic potential functions, commonly referred to as machine learning potentials (MLPs). These approaches use large datasets of structure–energy–force relationships generated by first-principles calculations such as DFT to fit high-dimensional potential energy surfaces in a data-driven manner, thereby achieving near-first-principles accuracy with computational efficiency approaching that of classical empirical potentials. MLPs include Behler–Parrinello neural network potentials, Gaussian approximation potentials (GAPs), spectral neighbor analysis potentials (SNAPs), moment tensor potentials (MTPs), and NequIP. Behler–Parrinello neural network potentials use atom-centered symmetry functions to describe local chemical environments and decompose the total energy into atomic energy contributions. They are suitable for fitting complex nonlinear potential energy surfaces, but in multicomponent systems the descriptors and network size become increasingly complex as the number of elements increases. GAP commonly combines local structural descriptors such as SOAP with Gaussian process regression, providing high accuracy and some uncertainty-estimation capability, but its computational cost is relatively high for large datasets and multicomponent systems. SNAP uses bispectrum components to represent local atomic environments and offers good computational efficiency and interpretability. Zhang et al. constructed a SNAP-based machine learning force field for ZrB_2_ and successfully predicted its structural, elastic, phonon, thermal-expansion, and thermal-transport properties, with applicability extended to 2500 K. This demonstrates that SNAP-type machine learning force fields (MLFFs) can be used for atomistic simulations of UHTCs at extreme temperatures [196].

MTP uses moment tensors to describe local environments and provides systematically expandable polynomial basis functions with high computational efficiency, making it suitable for combination with active learning in phase-space sampling. NequIP can achieve high-accuracy force and energy prediction with relatively small training datasets, but its training and inference costs are generally higher than those of linear or shallow-descriptor potentials. For HE-UHTCs, the highly complex local environments caused by random occupation by multiple principal elements mean that the key challenge for machine learning interatomic potentials (MLIPs) is not only fitting error but also transferability across composition, temperature, and defect structures. Liu et al. recently constructed a transferable neuroevolution potential (NEP) for high-entropy carbides. Using unary and binary carbide training data, they realized MD prediction of mechanical and thermal properties for 4–8 component high-entropy carbides and validated the results against first-principles and experimental data. This indicates that MLIPs for HE-UHTCs should focus on training-set coverage, representativeness of local environments, and cross-composition transferability [197].

**Figure 9:**
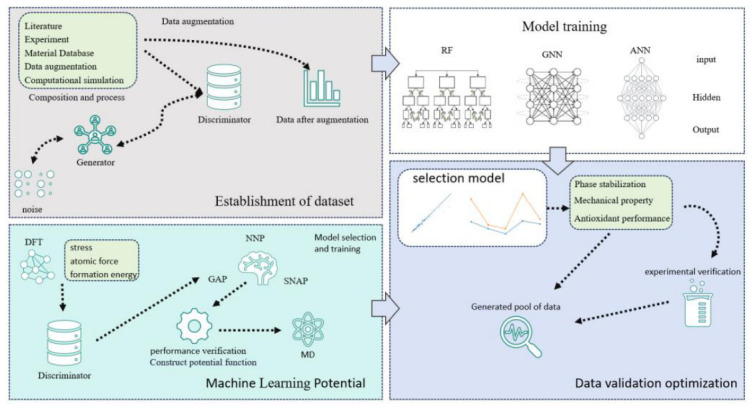
Flowchart of machine learning applications in high-entropy ceramics.

In recent years, ML has progressively evolved from an auxiliary screening tool into one of the core methods driving the rational design of HE-UHTCs. Unlike conventional design routes that rely heavily on empirical intuition and expensive first-principles calculations, ML can rapidly identify high-potential candidate systems across immense compositional spaces by constructing quantitative mappings among composition, processing, and performance. Figure 9 shows the application of machine learning in high entropy ceramics.

Particularly in high-entropy nitride and carbide systems, researchers have proposed ML-based design strategies built on low-cost descriptors, using readily accessible physicochemical parameters such as average atomic radius, mixing enthalpy, valence electron concentration, and electronegativity difference as input features. These models have achieved high-accuracy prediction of key mechanical properties, including hardness and elastic modulus [198]. Such approaches effectively circumvent the limitations of first-principles calculations in handling the large configurational space of high-entropy systems and provide a feasible route for screening multicomponent UHTCs. More importantly, some studies have coupled ML prediction with experimental synthesis to establish a closed-loop design framework of prediction–synthesis–validation–relearning, successfully identifying high-entropy ceramic materials with properties significantly superior to those of conventional systems within a limited number of iterations. This data-driven design paradigm is widely regarded as a crucial means of overcoming both the vast compositional space and the high experimental cost associated with HE-UHTCs [199].

In high-entropy nitrides and related coating systems, ML has further revealed the nonlinear relationships between compositional complexity and mechanical performance. Studies have shown that nitrogen content, the fraction of strong nitride-forming elements such as Al, Cr, Ta, and Nb, and deposition-processing parameters jointly determine the critical conditions for the transition of high-entropy nitrides from amorphous states to single-phase FCC solid solutions. This transition directly corresponds to a marked increase in hardness and elastic modulus [200].

Using interpretable models such as random forests combined with SHAP analysis, researchers have found that nitrogen content and substrate bias occupy dominant roles in hardness prediction for high-entropy nitrides, whereas the introduction of weak nitride-forming elements such as Ni and Mn significantly weakens solid-solution strengthening [200]. These findings provide clear physical guidance for compositional design of HE-UHTCs in coating applications: by strengthening nitride bonding and suppressing weakly bonded constituents, it is possible to achieve synergistic optimization of high hardness and high thermal stability. The combination of ML with high-throughput experiments has further demonstrated the power of data-driven approaches in establishing quantitative relationships among composition, processing parameters, and hardness in superhard ceramics [200].

### 6.2. Multiscale Modeling for the Design of UHTCs

**Figure 10:**
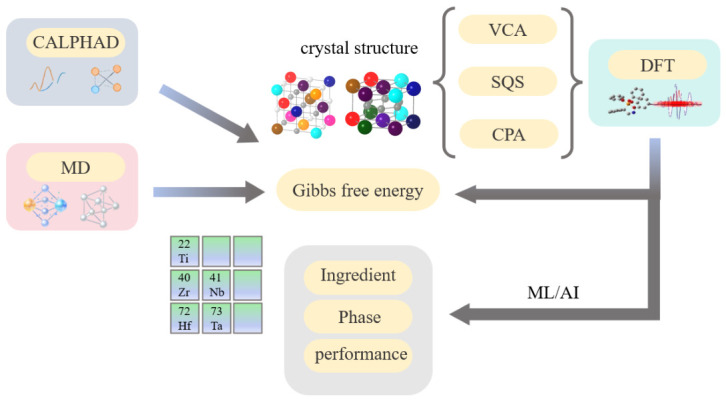
Computational design framework for high-entropy ceramics.

Multiscale modeling provides a foundational framework for understanding and predicting the behavior of UHTCs across different length and time scales, spanning electronic structure to macroscopic performance. In 2011, the establishment of the Materials Genome Initiative [201,202] promoted the integration of high-efficiency computation, advanced experimentation, and big data throughout the entire chain of materials design, manufacturing, and application. High-throughput computation (HTC) has become not only a computational strategy for resource reallocation but also an efficient route for materials design and virtual screening, whereby promising candidates can be identified from a vast materials space through computation [203]. For example, an HTC framework combining Taylor expansion and DFT calculations has been employed to predict the thermoelastic properties of UHTCs [204]. High-throughput DFT calculations have also been used to investigate the structural, elastic, and thermodynamic properties of solid solutions relevant to high-temperature UHTC applications [191]. Figure 10 shows the calculation and design route for high entropy ceramics.

At the atomic scale, first-principles calculations based on DFT have been widely used to evaluate intrinsic properties such as phase stability, bonding characteristics, elastic constants, and defect energetics. These calculations provide critical insights into the thermodynamic and mechanical behavior of UHTCs and serve as reliable sources of high-accuracy data for subsequent modeling efforts. In the design of high-entropy ceramic materials, DFT and MD simulations are commonly employed to evaluate Gibbs free energy and related thermodynamic quantities [205,206]. The material point method (MPM) has also been used to investigate microscale elastic deformation and damage evolution [207].

DFT and MD are both effective for probing atomic-scale phenomena, although they differ substantially in scope and precision. DFT calculations are typically limited to smaller systems but offer higher accuracy, whereas MD simulations can address larger systems and are therefore more suitable for materials with greater compositional complexity. In compositional design of high-entropy ceramics, first-principles calculations based on DFT are widely used to determine fundamental material parameters [208,209], while crystal structures are often modeled using special quasirandom structures (SQS), the coherent potential approximation (CPA), and the virtual crystal approximation (VCA). The VCA does not explicitly account for interactions among different elements. The SQS approach, although computationally more demanding and still limited in representing intrinsic atomic characteristics, incorporates the influence of the local atomic environment and is therefore generally more realistic than VCA. The CPA accounts for interactions among all constituent atoms and often yields predictions close to experiment, although it neglects local lattice distortion and is therefore limited in defect analysis [210].

At the mesoscale, thermodynamic modeling approaches such as CALPHAD and phase-field simulations are employed to predict phase equilibria, solid-solution stability, and microstructural evolution at elevated temperatures. These methods enable the assessment of multiphase interactions, grain growth behavior, and oxidation-induced phase transformations, all of which are critical to the long-term stability of ultra-high-temperature ceramics in extreme environments [211]. At the macroscopic scale, finite element modeling is commonly used to simulate thermal stresses, oxidation damage, and ablation behavior.

DFT or SQS/partial occupation (POCC) calculations can provide fundamental thermodynamic and mechanical information, including formation enthalpy, distance from the convex hull, elastic constants, electronic structure, local bonding, and energy distributions of disordered configurations. These data can be used to evaluate the phase stability of high-entropy ceramics. For example, Qureshi et al. used a free-energy model ΔG=ΔH−TΔS that combines DFT-calculated enthalpy relative to competing phases on the convex hull with ideal mixing entropy to predict the single-phase stability of high-entropy carbides and borides. When Delta ΔG<0, a single phase is predicted to form; when ΔG>0, multiphase decomposition is favored, and a 20 meV·atom^−1^ buffer region is used to represent model uncertainty. DFT data can also be transferred to machine learning potential functions to construct transferable MLIP or NEP energy surfaces, thereby extending atomic-scale energy, force, and stress information to larger length scales and higher-temperature MD simulations [212]. Liu et al. used high-entropy carbides as an example and trained a transferable neuroevolution potential using DFT data from unary and binary carbides. They then applied it to MD simulations of 4–8 component HECs to predict elastic constants, bulk modulus, shear modulus, tensile strength, lattice thermal conductivity, coefficient of thermal expansion, and melting point. This work shows that DFT training data can be converted through MLIP-MD into finite-temperature predictions of mechanical and thermophysical properties. Macroscopic processing parameters can also be transferred to mesoscale models [197]. In a study of chemical vapor deposition (CVD) growth of 2D materials, Momeni et al. fed temperature fields, gas-flow velocities, precursor concentration distributions, and deposition rates calculated by reactor-scale finite element modeling (FEM) into a phase-field model to predict island morphology, size, and spatial distribution, and validated the FEM-phase-field linkage experimentally. This strategy is also instructive for UHTCs: CALPHAD or DFT can provide phase stability and reaction driving forces; MD/MLIP can provide diffusion coefficients, thermal conductivity, elastic constants, and local distortion; phase-field models can describe oxide scale growth, phase decomposition, and microstructural evolution; and FEM can further receive these temperature-dependent parameters to calculate thermal stress, thermal-shock damage, ablation degradation, and component-level service reliability [213]. Therefore, future computational design of UHTCs should emphasize coupling among multiple computational methods [53].

AI and ML are transforming UHTC research from experimentally driven exploration toward data-assisted rational design. Nevertheless, their effectiveness ultimately depends on the integration of high-fidelity physical models, high-quality datasets, and realistic experimental constraints. In the future, the role of AI in UHTC research will extend beyond property prediction toward inverse design, multiscale coupling, and service-condition-aware models. Only through the deep integration of physics-informed machine learning, active learning, and high-throughput experimentation can the reliable design and engineering deployment of UHTCs in extreme environments truly be realized.

## 7. Conclusions and Future Perspectives

Although substantial progress has been made in compositional design, mechanical properties, oxidation/ablation resistance, and data-driven research on UHTCs, their engineering application remains constrained by several key scientific questions. Future work should not remain at the generic level of developing new compositions or introducing machine learning, but should instead prioritize the following three specific questions.

First, can the high-entropy effect persist during real high-temperature service? At present, the phase stability of HE-UHTCs is often evaluated based on room temperature characterization or ideal thermodynamic calculations. However, during sintering, cooling, oxidation, and long-term service, short-range order, elemental segregation, selective oxidation, and defect evolution may reduce local configurational entropy and even weaken entropy stabilization. Therefore, in situ high-temperature characterization, DFT, CALPHAD, MD, and oxidation experiments should be combined to clarify the competition among entropy stabilization, enthalpy-driven decomposition, and defect evolution [54,120,212].

Second, how can quantitative co-design rules be established among strength, toughness, thermal conductivity, and oxidation resistance and ablation resistance. Existing studies often optimize hardness, toughness, or oxidation resistance separately. In practical thermal protection system (TPS) components, however, materials simultaneously experience high heat flux, thermal stress, oxidation, aerodynamic shear, and mechanical loading, so improvement in a single property may compromise other key metrics [38,87,88,91,92]. Therefore, future work should establish a multi-objective evaluation framework that includes grain size, porosity, phase composition, interfacial bonding, oxide-scale viscosity, thermal conductivity, and residual strength, rather than relying only on room temperature hardness or single-test ablation rate.

Third, can current ML models move beyond fitting literature data toward reliable prediction under service conditions and effective inverse design? Current UHTC databases commonly suffer from small sample sizes, inconsistent testing conditions, missing microstructural variables, and insufficient external validation, all of which limit model generalization [184,188,192]. Future work should build standardized and high-fidelity databases and incorporate thermodynamic stability, bonding descriptors, diffusion mechanisms, oxide-scale evolution, and experimental uncertainty into physics-constrained machine learning frameworks to achieve integrated prediction from composition screening to processing, microstructure, properties, and service life. Furthermore, inverse design should become a core development direction. Starting from target service requirements, such as phase stability, strength-toughness synergy, oxidation, ablation resistance, tunable thermal conductivity, and low density, it should search backward for candidate compositions, microstructures, and processing windows. By integrating DFT, CALPHAD, MD, high-throughput experiments, active learning, and multi-objective optimization, a materials design framework linking target properties, descriptors, composition, processing, and experimental validation can be established, promoting the transition of UHTCs and HE-UHTCs from empirical screening to service-scenario-oriented efficient design.

## Data Availability

No new data were created or analyzed in this study.
